# BLMP-1/Blimp-1 Regulates the Spatiotemporal Cell Migration Pattern in *C. elegans*

**DOI:** 10.1371/journal.pgen.1004428

**Published:** 2014-06-26

**Authors:** Tsai-Fang Huang, Chun-Yi Cho, Yi-Ting Cheng, Jheng-Wei Huang, Yun-Zhe Wu, Athena Yi-Chun Yeh, Kiyoji Nishiwaki, Shih-Chung Chang, Yi-Chun Wu

**Affiliations:** 1 Institute of Molecular and Cellular Biology, National Taiwan University, Taipei, Taiwan; 2 Department of Bioscience, Kwansei Gakuin University, Gakuen, Sanda, Japan; 3 Department of Biochemical Science and Technology, National Taiwan University, Taipei, Taiwan; 4 Department of Life Science, National Taiwan University, Taipei, Taiwan; 5 Center for Systems Biology, National Taiwan University, Taipei, Taiwan; 6 Research Center for Developmental Biology and Regenerative Medicine, National Taiwan University, Taipei, Taiwan; 7 Institute of Atomic and Molecular Sciences, Academia Sinica, Taipei, Taiwan; University of California San Diego, United States of America

## Abstract

Spatiotemporal regulation of cell migration is crucial for animal development and organogenesis. Compared to spatial signals, little is known about temporal signals and the mechanisms integrating the two. In the *Caenorhabditis elegans* hermaphrodite, the stereotyped migration pattern of two somatic distal tip cells (DTCs) is responsible for shaping the gonad. Guidance receptor UNC-5 is necessary for the dorsalward migration of DTCs. We found that BLMP-1, similar to the mammalian zinc finger transcription repressor Blimp-1/PRDI-BF1, prevents precocious dorsalward turning by inhibiting precocious *unc-5* transcription and is only expressed in DTCs before they make the dorsalward turn. Constitutive expression of *blmp-1* when BLMP-1 would normally disappear delays *unc-5* transcription and causes turn retardation, demonstrating the functional significance of *blmp-1* down-regulation. Correct timing of BLMP-1 down-regulation is redundantly regulated by heterochronic genes *daf-12*, *lin-29*, and *dre-1*, which regulate the temporal fates of various tissues. DAF-12, a steroid hormone receptor, and LIN-29, a zinc finger transcription factor, repress *blmp-1* transcription, while DRE-1, the F-Box protein of an SCF ubiquitin ligase complex, binds to BLMP-1 and promotes its degradation. We have therefore identified a gene circuit that integrates the temporal and spatial signals and coordinates with overall development of the organism to direct cell migration during organogenesis. The tumor suppressor gene product FBXO11 (human DRE-1 ortholog) also binds to PRDI-BF1 in human cell cultures. Our data suggest evolutionary conservation of these interactions and underscore the importance of DRE-1/FBXO11-mediated BLMP-1/PRDI-BF1 degradation in cellular state transitions during metazoan development.

## Author Summary

The migratory path of DTCs determines the shape of the *C. elegans* gonad. How the spatiotemporal migration pattern is regulated is not clear. We identified a conserved transcription factor BLMP-1 as a central component of a gene regulatory circuit required for the spatiotemporal control of DTC migration. BLMP-1 levels regulate the timing of the DTC dorsal turn, as high levels delay the turn and low levels result in an early turn. We identify and characterize upstream regulators that control BLMP-1 levels. These regulators function in two ways, i.e. by destabilization of BLMP-1 through ubiquitin-mediated proteolysis and by transcriptional repression of the *blmp-1* gene to down-regulate BLMP-1. Interestingly, *blmp-1* also negatively controls these regulators. Our data suggest that a dietary signal input acts together with a double-negative feedback loop to switch DTCs from the “*blmp-1*-on” to the “*blmp-1*-off” state, promoting their dorsal turn. Furthermore, we show that some protein interactions in the circuit are conserved in *C. elegans* and humans. Our work defines a novel function of the conserved *blmp-1* gene in the temporal control of cell migration, and establishes a gene regulatory circuit that integrates the temporal and spatial inputs to direct cell migration during organogenesis.

## Introduction

Cell migration is important for organogenesis and development of animals. Numerous extracellular guidance cues and receptors for the spatial control of cell migration have been identified and characterized [1]. However, little is known about the temporal regulation of cell migration and how the spatial and temporal signals are coordinated to generate a specific and reproducible pattern of cell migration during development.

**Figure 1:**
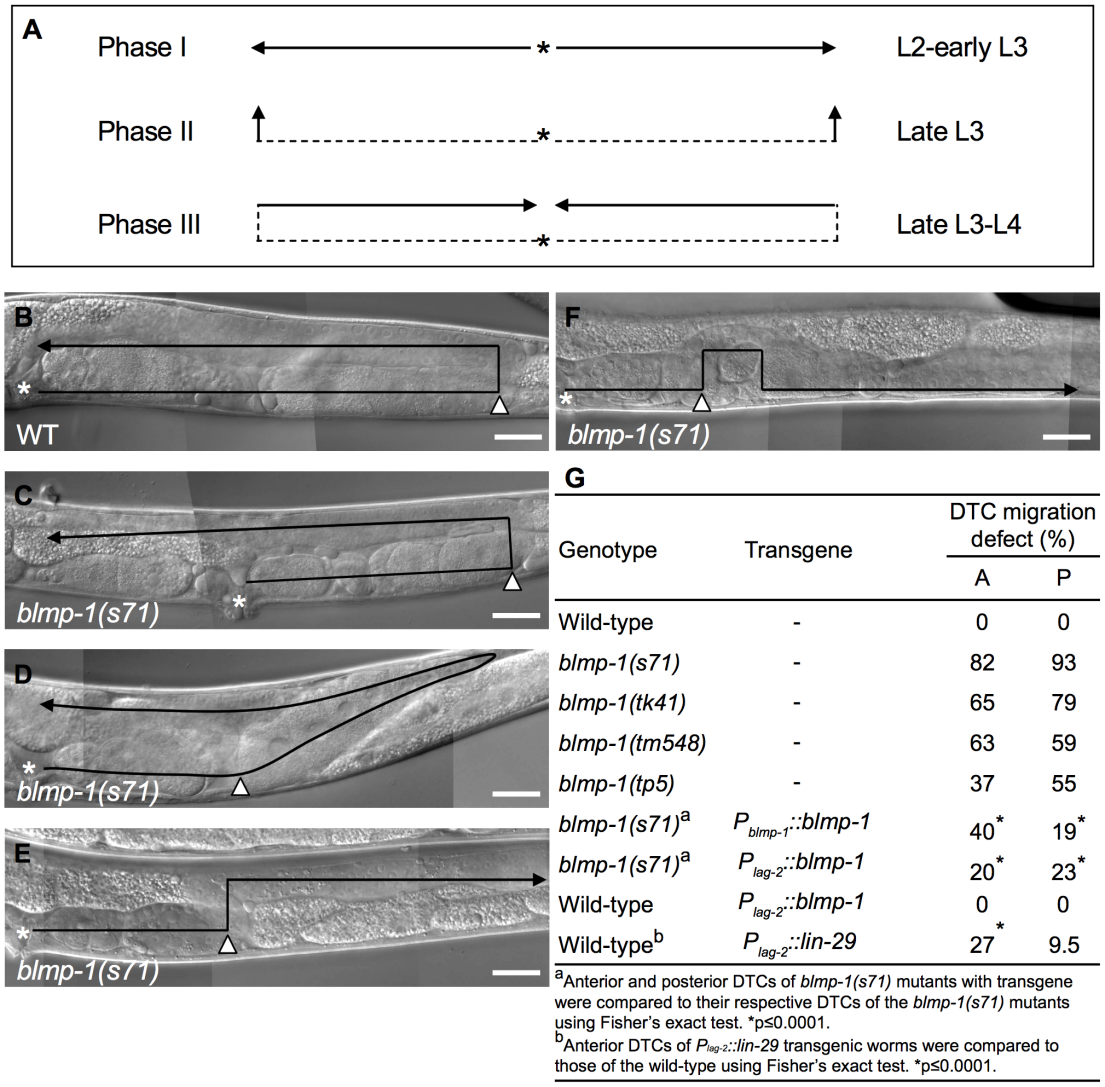
DTC migration defects of *blmp-1* mutants. (A) Schematic diagram showing the path and direction of DTC migration in phases I, II, and III. The developmental stage in which each migration phase occurs is indicated on the right. The solid line and arrow show, respectively, the path and direction of each migration phase. (B–F) DIC images of adult wild-type (B) and *blmp-1(s71)* (C–F) gonads. The black line shows the migratory path, starting from the asterisk, the DTC is indicated by the black arrowhead, and the white arrowhead indicates the position at which the DTC initiated dorsal phase II migration. In (C–F), the DTC had a shorter phase I migratory path (distance between the asterisk and white arrowhead) than the wild-type DTC (B); in addition, the DTC in (C) had a longer phase III migration path, that in (D) had a slanted phase II migration path (see text for details), and that in (E) moved in the opposite direction during phase III migration. (F) The DTC had a similar migratory trajectory to that shown in (E), except that it failed to stay on the dorsal side during phase III migration. The gonadal arm shown in B–E is posterior, while that in F is anterior, as the defect was only observed in this arm, as shown in Table S1. The picture in F was flipped 180 degrees so that its migration trajectory could be easily compared to those in B–E. Scale bar 40 µm. (G) Percentage of the indicated *blmp-1* mutants and transgenic worms with a DTC migration defect (shown in Figure 1C–F). A: anterior DTC, P: posterior DTC. At least 50 worms were scored for each genotype.

The bilobed gonad of *C. elegans* hermaphrodites develops from a four-cell primordium positioned in the ventral midbody [2]. The shape of the two symmetrical U-shaped gonadal arms is determined by the migratory paths of the two distal tip cells (DTC), leader cells found at the tip of each arm [3]. The DTCs undergo three sequential phases of migration and re-orient twice during the three larval developmental stages, thus providing a paradigm for the study of the spatio-temporal regulation of cell migration *in vivo* [2], [3]. In phase I during the L2 and early L3 stages, the anterior and posterior DTCs move centrifugally along the ventral body wall muscles towards the head or tail, respectively (Figure 1A). In phase II, they turn 90 degrees and move from the ventral to the dorsal muscles, then, during phase III, they again turn 90 degrees and move centripetally along the dorsal body wall muscles and halt in the mid-body. Both orthogonal turns occur during the late L3 stage. The timing of these turns is regulated by a redundant function of the heterochronic genes *daf-12*, *dre-1*, and *lin-29* [4]. A single mutation in any of these three genes has no effect on DTC migration, but mutation of 2 or 3 of the genes delays the L3-specific DTC migration pattern, which fails to take place even in L4 or the adult. *lin-29*, *daf-12*, and *dre-1* encode, respectively, a zinc-finger transcription factor, a steroid hormone receptor similar to the vertebrate vitamin D and liver-X receptor, and an F-Box protein of an SCF ubiquitin ligase complex [4]–[6], indicating that a complex mechanism, involving steroid hormone signaling, gene transcription, and protein degradation, is responsible for the temporal control of the dorsal turn. However, how these three genes function to do so is unclear.

The dorsal migration of DTCs is regulated by the guidance receptors UNC-5 and UNC-40 (a homolog of Deleted in Colorectal Cancer) [7]–[9], which drive DTCs to move away from the ventrally localized UNC-6 to the dorsal side [10], [11]. Dorsally localized UNC-129/TGF-β also promotes DTC dorsal migration through UNC-5 and UNC-40 receptors [12]. Mutations in these genes disrupt the ventral-to-dorsal migration of DTCs. *unc-40* appears to be transcribed in the DTCs throughout their migration [7], whereas *unc-5* is transcriptionally up-regulated at the time when the dorsal turn is initiated [13]. Precocious expression of *unc-5* cDNA at the time when DTCs have not yet turned dorsalward induces *unc-6*-dependent precocious dorsal migration [13]. These data show that *unc-5* is both necessary and sufficient for DTC dorsal migration and that the increase in *unc-5* transcription is responsible, at least in part, for the initiation of DTC dorsal migration [8], [9], [13]. However, how *unc-5* is temporally regulated to direct dorsal migration precisely at the late L3 stage is unclear.

In this study, we performed genetic screening for mutants defective in DTC migration and isolated *blmp-1* mutants. Our results showed that *blmp-1* is a heterochronic gene that acts with *daf-12*, *dre-1*, and *lin-29* in a regulatory circuit to control the correct timing of DTC migration. We also showed that this regulatory circuit is, at least in part, conserved in *C. elegans* and humans.

## Results

### *blmp-1* mutants show defective DTC migration

To identify genes that are important for the spatiotemporal regulation of DTC migration, we performed genetic screening for mutants defective in DTC migration, as described in the Materials and Methods, and isolated alleles *tp5* and *tk41*. A genetic complementation test showed that *tp5* and *tk41* were allelic, and that either allele failed to complement the previously identified mutation *dpy-24(s71)* (*dpy* stands for dumpy, shorter than wild-type) [14].

We mapped *dpy-24(s71)* to chromosome I, near *stp124*, by sequence tag site (STS) mapping [15] (Figure S1A). Three factor mapping using *unc-40* and *unc-75* and subsequent SNP mapping positioned *dpy-24(s71)* within the region between cosmids F45H11 and F37D6. Cosmids covering this region were microinjected into the *dpy-24*(*s71*) mutant, and cosmid F25D7 rescued the DTC migration defect (Figure S1B). The genomic DNA fragments corresponding to the 5 predicted open reading frames of F25D7 were individually amplified by long PCR and tested for their ability to rescue the *dpy-24*(*s71*) mutant and only F25D7.3, which contained a single *blmp-1* gene, had a rescue effect (Figure S1B). In addition, F25D7.3 RNA interference (RNAi) phenocopied the *dpy-24*(*s71*) mutant (Table 1). These results demonstrated that F25D7.3 corresponded to the *blmp-1* gene (named for its sequence similarity to mouse Blimp-1, see below) [16].

**Table 1:** Genetic interactions between *blmp-1* and the heterochronic genes *lin-29*, *dre-1*, and *daf-12* in the timing of the DTC dorsal turn.

Genotype	Dorsal turn^a^ (%)		
	Wild-type	Premature	Retarded
*blmp-1(s71)*	7	93	0
*blmp-1(RNAi)*	18	82	0
*lin-29(n546)*	100	0	0
*lin-29(RNAi)*	100	0	0
*dre-1(dh99)*	100	0	0
*daf-12(rh61rh411)*	100	0	0
*lin-29(n546); dre-1(dh99)*	0	0	100
*lin-29(n546); daf-12(rh61rh411)* b	0	0	97
*dre-1(dh99); daf-12(rh61rh411)* b	0	0	98
*blmp-1 (s71); lin-29(RNAi)* c	30	54	-
*blmp-1(s71); dre-1(dh99)*	27	73	0
*blmp-1(s71); daf-12(rh61rh41)* c	43	31	-
*blmp-1(RNAi); lin-29(n546); dre-1(dh99)*	22	43	35
*blmp-1(s71); lin-29(RNAi); daf-12(rh61rh411)*	15	37	41
*blmp-1(s71); dre-1(dh99); daf-12(rh61rh411)*	12	73	15
*lin-29(RNAi); dre-1(dh99); daf-12(rh61rh411)*	0	0	100
*blmp-1(s71); lin-29(RNAi); dre-1(dh99); daf-12(rh61rh411)*	17	43	40

a Percentage of animals in which the posterior DTCs showed a wild-type, premature, or retarded dorsal turn. At least 50 worms were scored for each genotype. Only the data for the posterior DTCs are shown.

b Posterior DTCs with a path-finding defect were seen in 3% of *lin-29(n546); daf-12(rh61rh411)* worms and 2% of dre-1(dh99);daf-12(rh61rh411) worms.

c Posterior DTCs with no obvious dorsal phase II migration and with the reversal of migration direction in centripetal phase III movement were seen in 16% of *blmp-1 (s71); lin-29(RNAi)* and 26% of *blmp-1(s71)*; *daf-12(rh61rh41)* worms. This defect may be caused by abnormal pathfinding or retardation in phase II and III migration.

**Figure 2:**
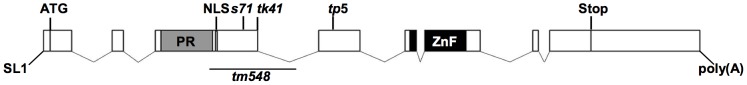
Gene structure of *blmp-1*. Gene structure of *blmp-1* deduced from genomic and cDNA sequences. The boxes indicate exons. The regions encoding the PRDI-BF1-RIZ1 homologous region (PR) domain, nuclear localization signal (NLS), and zinc finger motifs are marked, as is the *trans* spliced leader SL1. The positions of the *blmp-1* mutant alleles, including the region corresponding to the *tm548* deletion, are indicated.

The *blmp-1* gene encodes a protein with 27% identity to mouse B lymphocyte-induced maturation protein 1 (Blimp-1) and 26% identity to human positive regulatory domain I-binding factor (PRDI-BF1) (Figure S2). Both Blimp-1 and PRDI-BF1 are thought to act predominantly as transcription repressors and are essential for the terminal differentiation of B and T cells [17], [18]. As shown in Figure 2, BLMP-1, like Blimp-1 and PRDI-BF1, is predicted to contain a positive regulatory (PR) domain, a nuclear localization signal (NLS), and five Kruppel-type [(Cys)2-(His)2] zinc fingers. The zinc fingers of both Blimp-1 and PRDI-BF1 have been shown to bind to target DNA and are essential for their transcriptional repression activities [19]–[21].

Alleles *s71*, *tk41*, and *tp5* have, respectively, a non-sense mutation in codon 281, 381, or 434, and are predicted to encode truncated proteins without zinc fingers (Figure 2 and S2). The deletion allele *tm548*, which was isolated by a reverse genetic approach (National Bioresource Project), has an 810 bp deletion, removing part of exon 3 and part of intron 3 (Figure 2 and S2) and may result in a truncated BLMP-1 protein containing the first 254 amino acids of BLMP-1 and 17 amino acids encoded by the third intron.

### DTCs in *blmp-1* mutants undergo a precocious dorsal turn

The four *blmp-1* mutants, *s71*, *tk41*, *tp5*, and *tm548*, were found to have a similar set of defects, including a DTC migration abnormality (shown for *blmp-1(s71)* in Figure 1), a weak dumpy phenotype (shown for *blmp-1(s71)* in Figure S3A), and a partially penetrant embryonic lethality (shown for all four in Figure S3B). Wild-type *blmp-1* genomic DNA rescued the dumpy phenotype and embryonic lethality of the *blmp-1(s71)* mutant (Figure S3B), showing that these defects were caused by the loss of *blmp-1*.

The DTC migration patterns of these mutants were varied, but shared the common feature that the mutant DTCs had a shorter centrifugal phase I migration path and executed the dorsal turn at a point closer to the mid-body than wild-type DTCs (Figure 1B–G, Table S1), suggesting either slower movement and/or precocious initiation of the dorsal turn. However, we timed the movement of the DTCs during phase I migration and found that the *blmp-1* mutant DTCs did not migrate significantly slower than the wild-type DTCs (Table S2). In addition, some DTCs migrated obliquely with respect to the dorsal-ventral axis until they reached the dorsal muscle (Figure 1D); such a migratory route is probably due to the simultaneous execution of centrifugal phase I and dorsal phase II migrations, support for a precocious execution of the dorsal turn at the time when phase I migration normally occurs.

**Figure 3:**
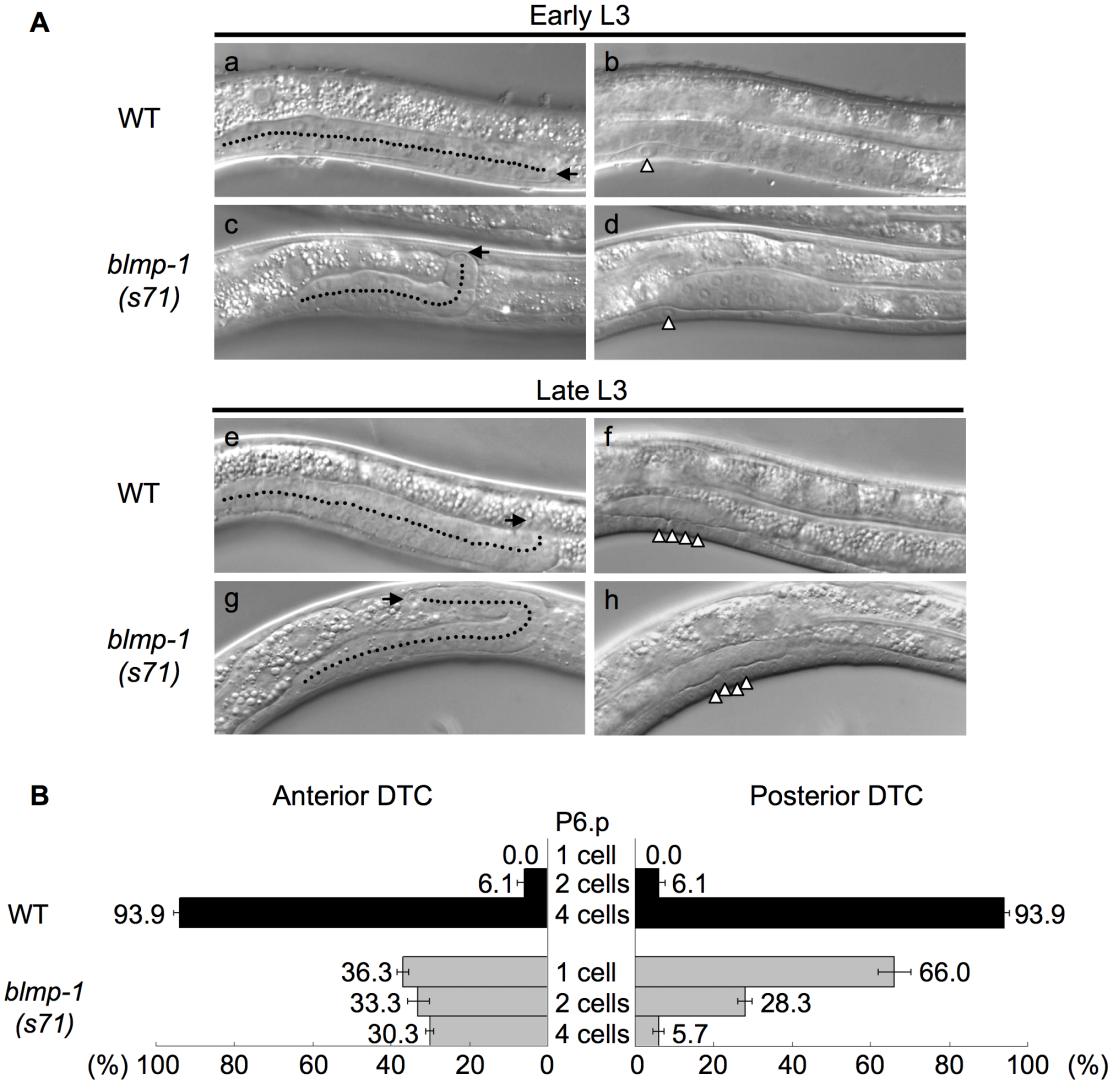
DTCs in the *blmp-1* mutant undergo a precocious dorsal turn. (A) DIC images of wild-type (WT) and *blmp-1(s71)* posterior gonadal arms during the early L3 (top panels) and late L3 (bottom panels) stages. (a–d) In early L3, when the worms were in the one-P6.p-cell stage (b, d), the wild-type DTC (a) was still in phase I migration, while the *blmp-1* DTC (c) had already made a dorsal turn. (e–h) In late L3, when the worms were in the four-P6.p-cell stage (f, h), the wild-type DTC (e) had just begun the dorsal turn, while the *blmp-1* DTC (g) had already completed the dorsal phase II migration and undergone centripetal phase III migration. In the left panel, the arrow and dotted line indicate, respectively, the DTC and its migratory path. In the right panel, the arrowhead indicates the P6.p division stage of the same worm shown in the left panel. (B) The percentage of worms with anterior (left) and posterior (right) DTCs that initiated the phase II dorsal turn at the indicated division stage of the P6.p cell (shown in the center) in wild-type (black bars) and *blmp-1(s71)* (gray bars) worms. At least 33 worms were examined for each genotype.

To examine whether the abnormal DTC migration pattern of the *blmp-1* mutants was indeed caused by a precocious dorsal turn, we performed a time-course analysis of DTC migration in the wild-type and *blmp-1(s71)* mutant, using the division stages of the vulval precursor cell P6.p as temporal developmental markers, as described previously [22]. P6.p is generated in mid L1, undergoes three rounds of cell division during L3, and gives rise to eight descendants that constitute the vulva [23]. Figure 3A shows representative DIC images of wild-type (a, b, e, f) and *blmp-1(s71)* (c, d g, h) posterior gonadal arms in early L3 (top panels) and late L3 (bottom panels). Figure 3B shows that, in the wild-type, the DTCs in more than 90% of worms underwent ventral-to-dorsal migration at the four-P6.p cell stage and the DTCs in none made a dorsal turn before P6.p cell division, whereas, in the *s71* mutant, the anterior DTCs in 36% of worms and the posterior DTCs in 66% of worms had turned dorsalward before P6.p divided. These results demonstrate that loss of *blmp-1* causes a precocious initiation of DTC dorsal migration (early L3, rather than late L3) and that *blmp-1* functions to prevent a precocious DTC dorsal turn.

### BLMP-1 is present in the DTC before the dorsal turn

**Figure 4:**
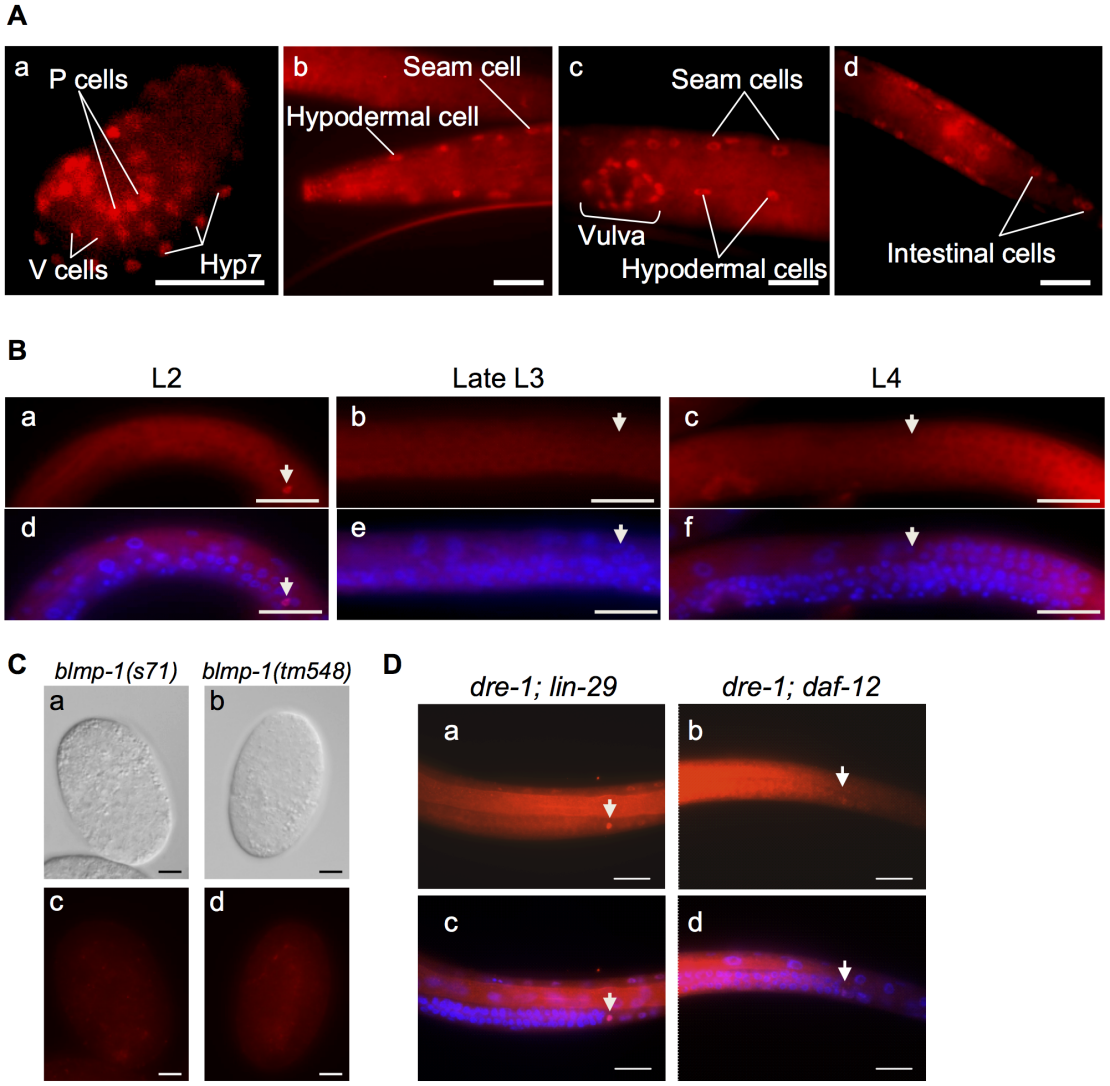
BLMP-1 expression pattern and its regulation. (A) Representative images of a wild-type embryo (a) or larva (b–d) stained with anti-BLMP-1 antibodies. (a) In the 1.5-fold embryo, BLMP-1 is detected in Hyp7, V cells, and P cell precursors. (b–d) In the larva, BLMP-1 is detected in hypodermal and seam cells (b and c), vulval cells (c), and posterior intestinal cells (d). Scale bar 40 µm. (B) BLMP-1 levels in wild-type DTCs at different larval stages revealed by immunostaining with anti-BLMP-1 antibody (a–c) or together with DAPI staining (d–f). (a) BLMP-1 is detected during centrifugal phase I migration in L2. (b, c) No BLMP-1 is detected during dorsal phase II migration in late L3 (b) or during centripetal phase III migration in L4 (c). (d, e, f) The same worms as those in, respectively, a, b, or c stained with DAPI to label nuclei and to examine the developmental stage of the gonad. Scale bar 20 µm. The arrowheads indicate DTCs. (C) The DIC (a, b) and immunostaining (c, d) images of *blmp-1(s71)* (a, c) and *blmp-1(tm548)* (b,d) embryos stained by anti-BLMP-1 antibodies. Scale bar 10 µm. (D) Presence of BLMP-1 at the L4 stage in the DTCs of the double mutants *lin-29;dre-1* (a) and *dre-1;daf-12* (b), as revealed by immunostaining with anti-BLMP-1 antibodies. (c, d) The same worms as those in a and b, respectively, stained with DAPI. Scale bar 20 µm.

To determine the localization of BLMP-1 and explore its function, we raised polyclonal antibodies against bacterially expressed recombinant BLMP-1 (Materials and Methods) and used the affinity-purified antibodies to stain whole-mount animals. The results showed that BLMP-1 was localized in the nucleus and was detected in hypodermal, vulval, and intestinal cells (Figure 4A), as well as DTCs (Figure 4B). We next stained the mutant embryos with the *blmp-1* mutations (*s71*, *tm548*, *tp5* and *tk71*), which are loss-of-function recessive alleles by genetic tests (Materials and Methods). Little or no signals were detected in these mutant embryos as shown in representative images in Figure 4C, demonstrating the specificity of the antibodies. Notably, BLMP-1 was seen in DTCs prior to the mid L3 larval stage (two P6.p-descent cells), but not during, or after, mid L3 stage, after the DTCs had undergone the dorsal turn (Figure 4B). This result and the precocious dorsal turn phenotype of the *blmp-1* mutant support a model in which BLMP-1 functions in DTCs before mid L3 stage to prevent these cells from undergoing a precocious dorsal turn.

The localization of BLMP-1 in DTCs supports the model that BLMP-1 controls the timing of DTC dorsal migration in a cell-autonomous fashion. We then tested whether expression of *blmp-1* cDNA in DTCs under the control of the *lag-2* promoter *P_lag-2_* is sufficient to rescue the DTC migration defect of the *blmp-1* mutant. *P_lag-2_* drives gene expression in DTCs, but not in body wall muscle or hypodermis, the structures on which DTCs migrate [24]. In *blmp-1* mutants carrying the *P_lag-2_ blmp-1* transgene, the percentage of worms with abnormal anterior or posterior DTC migration was reduced, respectively, from 82% to 20% and from 93% to 23% (Figure 1G), similar levels to those seen when *blmp-1* cDNA was expressed under the control of the *blmp-1* endogenous promoter *P_blmp-1_* (40% or 19% for the anterior and posterior DTC, respectively; Figure 1G). This result further supports a cell-autonomous role of *blmp-1* in DTC migration.

### *blmp-1* represses *unc-5* transcription to prevent the DTCs from dorsalward turning

Next, we tested whether *blmp-1* may prevent dorsalward turning of DTCs by regulating a dorsal-ventral guidance system at the early larval stage. The dorsal migration of DTCs is regulated by the guidance receptors UNC-5 and UNC-40 (a homolog of Deleted in Colorectal Cancer) [7]–[9], which drive DTCs to move away from the ventrally localized UNC-6 [10]–[11]. The observations that *blmp-1*, *unc-5* and *unc-40* function cell-autonomously in the control of DTC migration [7], [9] (Figure 1G) suggest that *blmp-1* may prevent DTC precocious dorsalward turning by regulating *unc-5* and/or *unc-40*. *unc-40* appears to be transcribed in the DTCs throughout their migration [7], whereas *unc-5* is transcriptionally up-regulated at the time when the dorsal turn is initiated [13]. Using the transgene *P_unc-5(1 kb)_ gfp*, in which an approximately 1 kb sequence upstream of the *unc-5* coding sequence was used to drive the GFP reporter, we confirmed this *unc-5* expression pattern, i.e. *unc-5* was expressed during and after, but not before, the DTC dorsal turn (Figure S4). Interestingly, this temporal pattern of *unc-5* transcription is complementary to that of BLMP-1 expression in DTCs, as BLMP-1 was present in DTCs only before the dorsal turn (Figure 4B). This raised the possibility that BLMP-1 inhibits *unc-5* transcription and thus prevents DTCs from turning dorsalward. If this were the case, the correct timing of the disappearance of BLMP-1 from DTCs would alleviate this transcriptional inhibition and allow *unc-5* transcription and the DTC dorsal turn. We tested this hypothesis by altering the temporal expression pattern of *blmp-1* and examining their effects on *unc-5* transcription.

**Figure 5:**
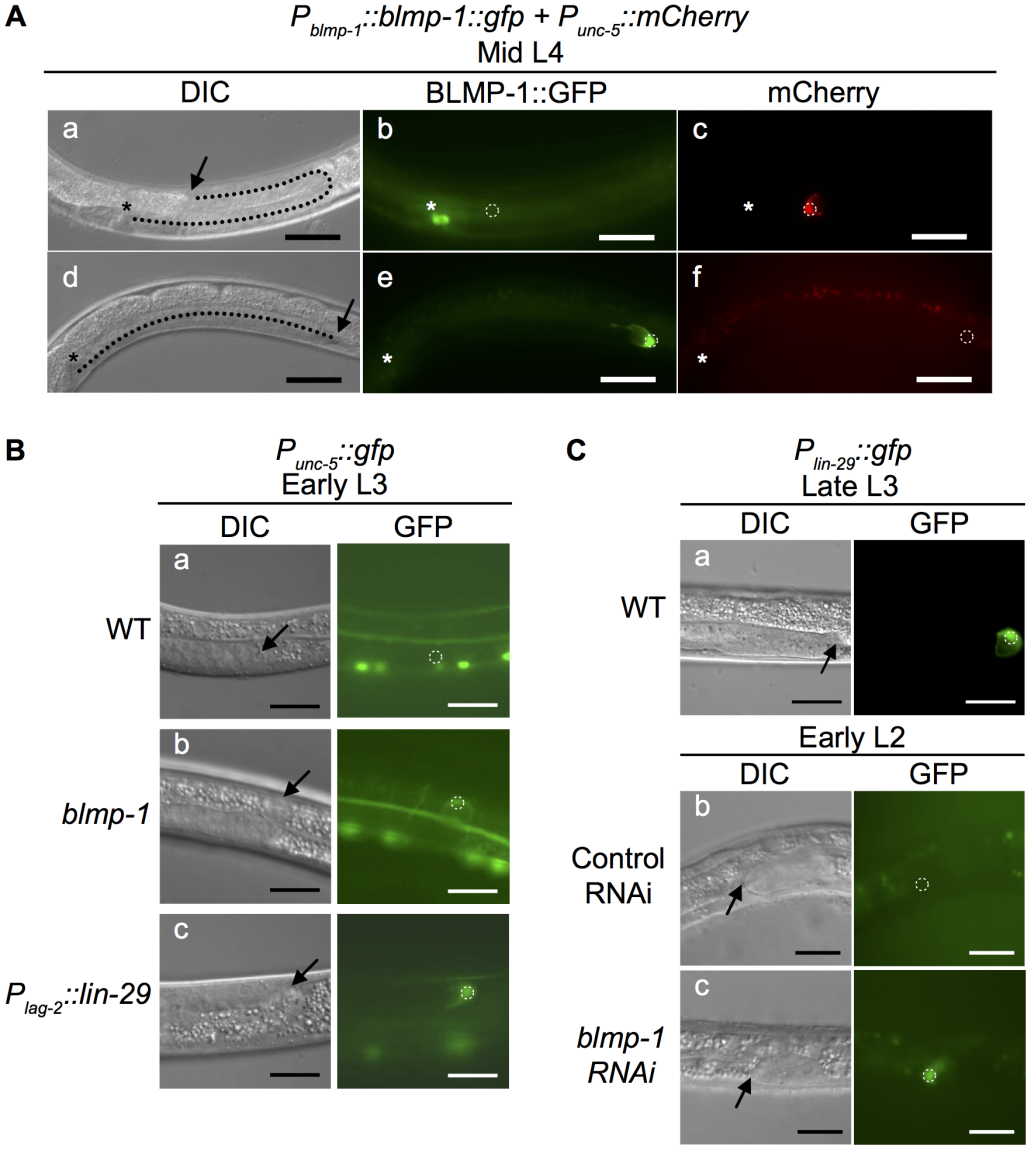
Effects of heterochronic mutations on the transcription of *unc-5* and *lin-29* in DTCs. (A) Persistent BLMP-1 expression in DTCs suppresses *unc-5* transcription during late larval development. DIC, GFP, and mCherry images at the mid L4 stage of two worms carrying the *P_blmp-1_::bmp-1::gfp* and *P_unc-5(4.6 kb)_::mCherry* transgenes. (a–c) A representative worm in which BLMP-1 was normally down-regulated. The DTC has undergone a dorsal turn (a) and does not express BLMP-1::GFP (b), but expresses *unc-5*. (d–f) A representative worm in which BLMP-1 was still expressed. The DTC has failed to turn dorsalward and is moving centrifugally (d), shows persistent BLMP-1::GFP expression (e), and does not express *unc-5* (f). The arrows indicate DTCs and the asterisks the developing vulva. Scale bar 40 µm. (B) The *blmp-1* mutation causes precocious *unc-5* transcription through precocious expression of *lin-29*. (a–c) DIC and GFP images of the posterior DTC at the early L3 stage in a wild-type worm (a), a *blmp-1(s71)* worm (b), and a *P_lag-2_::lin-29*-expressing worm (c). All worms carried a *P_unc-5(4.6 kb)_::gfp* transgene. Scale bar 20 µm. (C) DIC and GFP images of transgenic animals carrying *P_lin-29_::gfp* (a) during late L3 stage. The transgenic animal carrying *P_lin-29_::gfp* treated with control vector RNAi (b) or *blmp-1* RNAi (c) during the early L2 stage. The arrows indicate DTCs. Scale bars 20 µm.

At the early L3 stage when the P6.p cell has not yet divided, no wild-type worms contained DTCs that had turned dorsalward or showed any expression of the *P_unc-5(1 kb)_::gfp* transgene (Figure 5Ba), whereas, in some *blmp-1(s71)* mutants, DTCs at the same stage had performed the dorsal turn and displayed precocious *unc-5* transcription (Figure 5Bb). Thus, loss of *blmp-1* causes precocious *unc-5* expression, which coincides with the precocious dorsalward turning of DTCs.

Next, we tested whether the *blmp-1* precocious dorsal turn phenotype required *unc-5* by examining and comparing the DTC dorsal migration patterns of the *blmp-1* and *unc-5* single mutants and the *blmp-1; unc-5* double mutant. Approximately 48% anterior DTCs and 83% posterior DTCs failed to turn dorsalward in the *unc-5(e53)* mutant (Table S3). No precocious dorsal turn was observed in the *blmp-1 (s71); unc-5* double mutant, showing that the *unc-5(e53)* mutation blocked the precocious dorsal turn phenotype of the *blmp-1 (s71)* mutant and that the *blmp-1* precocious dorsal turn phenotype required *unc-5*.

### Constitutive expression of *blmp-1* delays dorsal turn and inhibits *unc-5* expression

Because BLMP-1 is present in the DTC before the dorsal turn, we examined the significance of *blmp-1* down-regulation and its effect on the initiation of DTC dorsalward turning. To this end, we overexpressed *blmp-1* using the transgene *P_blmp-1_::blmp-1::gfp*, in which the fusion protein BLMP-1::GFP was expressed under the control of the promoter *P_blmp-1_*. In the resulting transgenic line, 10% of transgenic worms had DTCs that displayed normal BLMP-1::GFP down-regulation, underwent a normal dorsal turn (Figure 5A, a, b), whereas 90% of transgenic worms showed persistent BLMP-1::GFP expression, even at the L4 stage, showing that BLMP-1::GFP was not appropriately down-regulated in these worms, and these worms showed no sign of dorsal turn (Figure 5A, d, e). This retarded turn phenotype was in contrast to the precocious dorsal migration phenotype caused by loss of *blmp-1* (Figures 1C–G). Collectively, these results show that the BLMP-1 level is important regulation for DTC dorsal migration and that the timely disappearance of BLMP-1 allows the DTCs to turn dorsalward, hence switching their migration phase from centrifugal phase I migration to ventral-to-dorsal dorsal phase II migration.

To test whether *blmp-1* overexpression might repress *unc-5* expression in DTCs and thus resulted in the retarded turning phenotype, we expressed the transgene *P_blmp-1_::blmp-1::gfp* in worms carrying the *P_unc-5(1 kb)_::mCherry* reporter. In the resulting transgenic line, 10% of transgenic worms had DTCs that displayed normal BLMP-1::GFP down-regulation, underwent a normal dorsal turn, and showed *P_unc-5(1 kb_*_)_::*mCherry* expression (Figure 5A, a–c), whereas 90% of transgenic worms showed persistent BLMP-1::GFP expression, and these worms showed no sign of dorsal turn or *unc-5* expression (Figure 5A, d–f). Thus, constitutive *blmp-1* expression in the late larval stage represses *unc-5* transcription and blocks the DTC dorsal turn. These results confirmed the causal relationship of the reciprocal patterns of BLMP-1 expression and *unc-5* transcription in DTCs and support the model in which BLMP-1 inhibits *unc-5* transcription and thus prevents DTCs from turning dorsalward during early larval development.

### Reciprocal suppression of *blmp-1* and either *daf-12*, *dre-1*, or *lin-29* in the control of the timing of DTC dorsal migration

The heterochronic genes *daf-12*, *dre-1*, and *lin-29* function redundantly to specify the temporal identity of DTCs and prevent DTCs from undergoing retarded ventral-to-dorsal migration [4]. We tested the genetic interaction of *blmp-1* with *daf-12*, *dre-1*, and *lin-29* in the temporal control of DTC dorsal turn. For *daf-12*, we used the null allele *rh61rh411*. Because complete loss of *dre-1* or *blmp-1* caused lethality [4], [25] and the *lin-29(n546)*; *blmp-1(s71)* double mutant was very sick and could not be maintained as homozygote, we used viable alleles and/or RNAi for *dre-1*, *blmp-1* and *lin-29* to analyze the genetic interactions of these genes. The precocious phenotype of the *blmp-1(s71)* mutant was partially suppressed by a single mutation of *lin-29*, *dre-1*, or *daf-12* or combined mutations of any 2 or all 3 (Table 1). For example, 93% of *blmp-1(s71)* mutants had a precocious DTC migration defect, whereas 54% of the *blmp-1(s71); lin-29(RNAi)* double mutants, 73% of the *blmp-1(s71); dre-1(dh99)* double mutants, and 31% of the *blmp-1(s71);daf-12(rh61rh41)* double mutants displayed a precocious turn defect. This suggests that the *blmp-1* precocious phenotype may require the activity of *lin-29*, *dre-1*, or *daf-12*, and that *blmp-1* may function upstream of, or in parallel with, *lin-29*, *dre-1*, and *daf-12* to prevent a precocious DTC dorsal turn during early larval development. Conversely, the *blmp-1* mutation also partially suppressed the retarded dorsal migration of the double or triple mutants of *lin-29*, *dre-1*, and *daf-12* (Table 1). For example, 98% of the *dre-1(dh99); daf-12(rh61rh411)* double mutants showed retarded DTC migration, whereas 12%, 73%, and 15% of the *blmp-1(71); dre-1(dh99); daf-12(rh61rh411)* triple mutants showed, respectively, wild-type, precocious, or retarded DTC migration. This suggests that the retarded phenotype of the *lin-29*, *dre-1* and *daf-12* double and triple mutants may require *blmp-1* activity and that *lin-29*, *dre-1*, and *daf-12* may function upstream of, or in parallel with, *blmp-1* to prevent a delay in the DTC dorsal turn during the late larval stage. Thus, two distinct regulatory hierarchies in these heterochronic genes are likely employed to specify the temporal identities of DTCs at the early and late larval stages, and the switch from the “*blmp-1*-on” state to the “*blmp-1*-off” state during developmental progression may determine the timing of the DTC dorsal turn.

### BLMP-1 transcriptionally represses *lin-29* to prevent DTCs undergoing a precocious dorsal turn

The observation that mutation of *daf-12*, *dre-1*, or *lin-29* partially suppressed the *blmp-1*-related precocious DTC dorsal migration defect (Table 1) prompted us to examine whether *blmp-1* negatively regulated the transcription of *daf-12*, *dre-1*, or *lin-29*. Using chromatin immunoprecipitation coupled with high-throughput DNA sequencing (ChIP-seq), the modENCODE Consortium has shown that BLMP-1 binds to the upstream sequence of *lin-29*, but not that of *daf-12* or *dre-1*, at the early larval stage [26]. This suggested that BLMP-1 might negatively regulate *lin-29* expression. To test this possibility, we analyzed and compared *lin-29* transcription in the wild-type and *blmp-1* mutants using the transcriptional reporter *P_lin-29_::gfp*, in which *gfp* was expressed under the control of the *lin-29* promoter *P_lin-29_* (Figure 5Ca). We confirm that the transcription of *lin-29* starts at approximately mid L3 stage and continues during L4 [4], [27]. We found that knockdown of *blmp-1* using RNAi resulted in precocious expression of *P_lin-29_::gfp* at the L2 stage (Figure 5Cb,c), showing that *blmp-1* represses *lin-29* transcription at the L2 stage.

To investigate the significance of this *blmp-1*-mediated *lin-29* down-regulation during DTC phase I migration in L2, we precociously expressed *lin-29* under the control of the *P_lag-2_* promoter using the *P_lag-2_::lin-29* transgene. In the wild-type animals carrying this transgene, 27% and 9.5% of the anterior or posterior DTCs, respectively, underwent a precocious dorsal turn (Figure 1G). This result shows the functional importance of *blmp-1*-mediated repression of *lin-29* transcription in preventing precocious DTC dorsal migration during early larval development.

### The heterochronic genes *daf-12*, *dre-1*, and *lin-29* act redundantly to down-regulate BLMP-1 levels in the mid and late L3 stages

The observation that the constitutive expression of *blmp-1* in DTCs throughout larval development prevented them from performing the dorsal turn (Figure 5A) highlights the importance of BLMP-1 down-regulation in promoting the dorsal turn. Two observations suggested that *daf-12*, *lin-29*, and *dre-1* might be responsible for BLMP-1 down-regulation. First, like worms constitutively expressing *blmp-1* (Figure 5Ad–f), the double and triple mutants of *daf-12*, *lin-29*, and *dre-1* had a retarded DTC migration phenotype (Table 1). Second, loss of *blmp-1* partially suppressed the retarded phenotype of the double and triple mutants (Table 1), showing that the defect in the double and triple mutants required *blmp-1* activity. We therefore examined BLMP-1 levels in mutants defective in *daf-12*, *lin-29* and/or *dre-1* using immunostaining with anti-BLMP-1 antibody.

As mentioned above, in the wild-type control, BLMP-1 was not detected in DTCs at the L4 stage (Figure 4B) and a similar result was observed in the *daf-12*, *dre-1*, and *lin-29* single mutants. In contrast, persistent BLMP-1 expression in L4 stage was seen in DTCs from the double mutants *dre-1(dh99); lin-29(n546)* (100% of 30 worms scored), *dre-1(dh99); daf-12(rh61rh411)* (20% of 60 scored), and *lin-29(RNAi); daf-12(rh61rh411)* (∼5% of 106 scored) (examples shown in Figure 4D). The intensity of the persistent BLMP-1 signal was much weaker in the *lin-29(RNAi); daf-12(rh61rh411)* double mutant than in the other double mutants and bleached too quickly to be photographed by our imaging system. The *daf-12(rh61rh411)* allele is molecular null, while *lin-29(n546)* and *dre-1(dh99)* are partial loss-of-function [4], [5]. These results suggest that DRE-1 acts together with transcription factor LIN-29 or, to a lesser extent, with transcription factor DAF-12 for efficient down-regulation of BLMP-1 and correct timing of the control of DTC dorsal migration.

### LIN-29 and DAF-12 repress *blmp-1* transcription to promote the DTC dorsal turn

**Figure 6:**
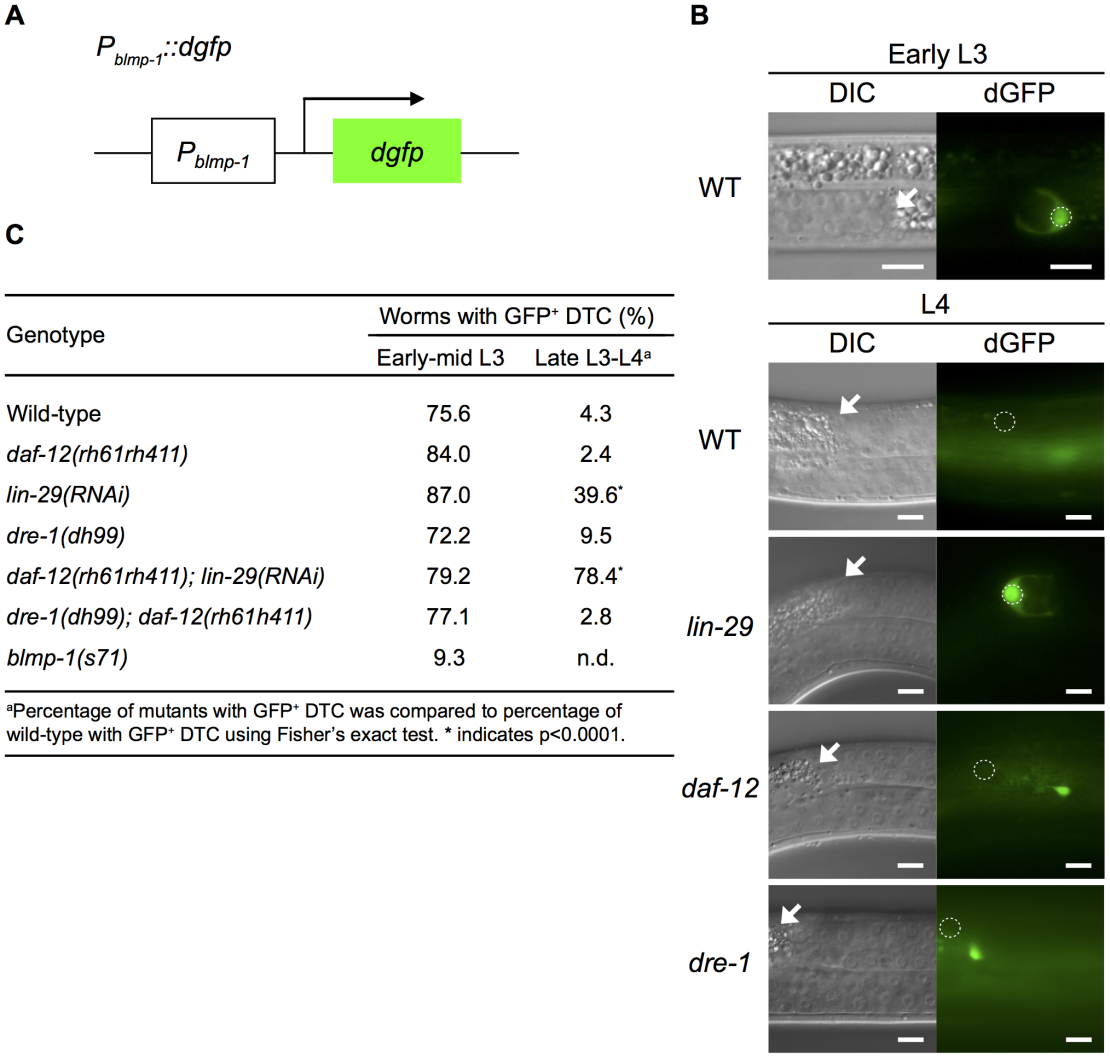
*blmp-1* is down-regulated at the transcriptional level by *lin-29* and *daf-12*. (A) Schematic diagram of the *P_blmp-1_::dgfp* transcriptional reporter. (B) DIC (left) and dGFP (right) images of worms carrying the *P_blmp-1_::dgfp* reporter in the indicated genotypes during the early L3 stage (upper panels) or L4 stage (lower panels). Scale bar 10 µm. (C) Percentages of worms with the posterior DTC expressing the *P_blmp-1_::dgfp* transgene (GFP^+^ DTC) at the indicated developmental stages. More than 20 worms were examined for each genotype at each developmental stage.

To determine how BLMP-1 was down-regulated by *lin-29*, *daf-29*, and *dre-1*, we used two *gfp* reporters in an *in vivo* expression assay to examine whether *blmp-1* expression was repressed at the transcriptional level by transcription factors LIN-29 and DAF-12 and at the post-transcriptional level by the F-Box protein DRE-1. We first generated a transcriptional reporter *P_blmp-1_::dgfp* (Figure 6A), in which dGFP (destabilized GFP) was controlled by the *P_blmp-1_* promoter. dGFP contains a PEST sequence and has a shorter half-life than normal GFP [28] and therefore allows sensitive detection of promoter activity. In animals carrying the integrated *P_blmp-1_::dgfp* transgene, the DTCs of 75.6% of worms expressed GFP in the early and mid L3 stages, while only 4.3% expressed GFP in the late L3 and L4 stages (Figure 6B and 6C), suggesting that *blmp-1* transcription is strongly repressed during, and after, late L3 stage.

Next, we tested whether *daf-12* or *lin-29* was responsible for *blmp-1* transcriptional repression by examining *P_blmp-1_::dgfp* transgene expression in the absence of *daf-12* and/or *lin-29*. As shown in Figure 6C, in early and mid L3, loss of either *daf-12* or *lin-29* did not affect the percentage of worms with DTCs expressing *dgfp*. However, during the late L3 and L4 stages, loss of *lin-29*, but not *daf-12*, increased the percentage of worms with DTCs expressing dGFP in late L3 and L4. For example, only 4.3% or 2.4% of wild-type or *daf-12* mutant worms, respectively, had DTCs expressing dGFP, whereas 39.6% of *lin-29*(RNAi) worms had dGFP-expressing DTCs. This result shows a differential requirement for *lin-29* (stronger) and *daf-12* (weaker) for *blmp-1* transcriptional repression during, and after, the late L3 stage (Figure 6C). In addition, 78.4% of the *daf-12(rh61rh411); lin-29(RNAi)* double mutants had DTCs expressing dGFP (Figure 6C). These results suggest that *daf-12* plays a non-essential, but auxiliary, role in the repression of *blmp-1* transcription.

We also tested the involvement of *dre-1* in *blmp-1* transcriptional repression. No effect on the *P_blmp-1_::dgfp* transcription level was observed when the *dre-1(dh99)* mutation was introduced into the wild-type or the *daf-12*mutant (Figure 6C). Thus, *dre-1* probably plays no role in *blmp-1* transcriptional repression. Interestingly, *blmp-1* seems to be required for its own expression. During early and mid L3 stages, approximately 75% of the wild-type worms had DTCs expressing dGFP from the *P_blmp-1_::dgfp* transgene, but only 9% of the *blmp-1(s71)* worms had DTCs expressing dGFP.

### DRE-1 functions in an SCF ubiquitin ligase complex to regulate BLMP-1 levels

Previous genetic and biochemical data have shown that the F-Box protein DRE-1 functions in an SCF ubiquitin ligase complex, which contains CUL-1 (a cullin scaffold protein), SKP-1 (a SKP1-like adaptor that binds to the F-Box protein), and RBX-1 (an RBX ring finger that bridges to the E2 ubiquitin conjugating enzyme), and is important for the temporal control of somatic and gonad development [4] and the timing of tail spike cell death [29]. This raised the possibility that DRE-1 may regulate BLMP-1 stability through ubiquitin-mediated proteolysis. Like *dre-1* RNAi, RNAi for *skp-1*, *rbx-1*, or *cul-1* caused a retarded DTC migration phenotype in the *daf-12(rh61rh411)* single mutant [4] (Table S4), consistent with a model in which DRE-1 targets protein(s) for proteolysis in an SCF ubiquitin ligase complex during the temporal regulation of the DTC dorsal turn.

**Figure 7:**
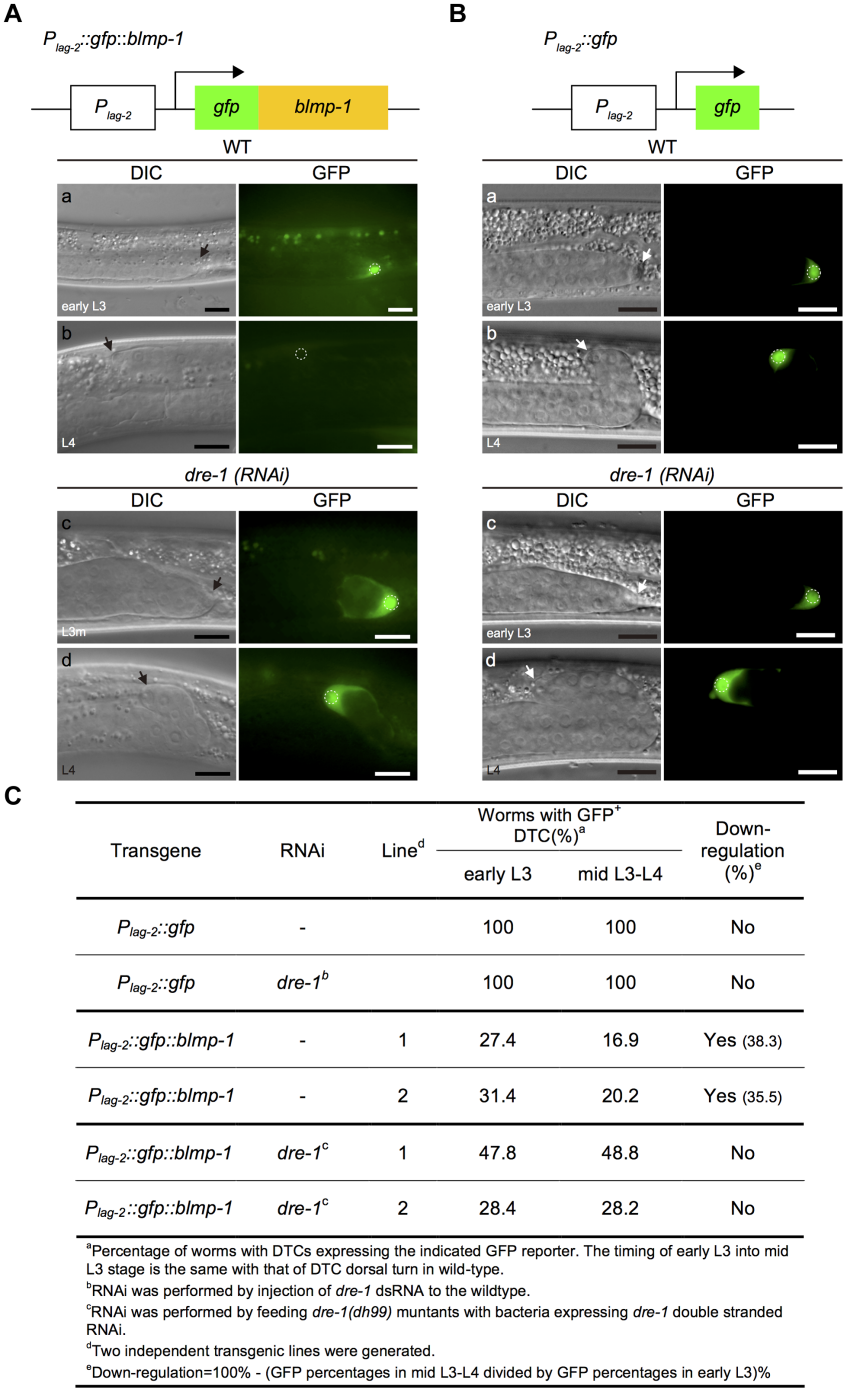
The stability of the GFP::BLMP-1 fusion protein is negatively regulated by *dre-1*. (A) Schematic diagram of the *P_lag-2_::gfp*::*blmp-1* construct. (a–d) DIC (left) and GFP (right) images of wild-type (WT) and *dre-1*(RNAi) worms carrying the *P_lag-2_::gfp*::*blmp-1* transgene during early L3 stage (top panels) or L4 stage (bottom panels). (B) Schematic diagram of the *P_lag-2_::gfp* construct. (a–d) DIC (left) and GFP (right) images of wild-type (WT; top panels) and *dre-1(RNAi)* (bottom panels) worms carrying the *P_lag-2_::gfp* transgene during early L3 stage (top) or L4 stage (bottom). L3m: L3 molt. In both A and B, the scale bars are 10 µm, the arrows indicate DTCs, and the DTC nuclei are circled in the GFP images. (C) Percentage of worms with DTCs expressing the indicated GFP reporter (GFP^+^ DTCs) in the transgenic animals at the indicated larva stage. More than 20 animals were examined in each genotype at each larval stage.

Using the transgene *P_lag-2_::gfp::blmp-1*, in which BLMP-1 was tagged with GFP and expressed under the control of the *P_lag-2_* promoter (Figure 7A), we next examined whether DRE-1 destabilized BLMP-1. Two independent transgenic lines carrying an extrachromosomal transgene array were generated. In line 1, approximately 27% of worms expressed GFP in DTCs during early L3 and the percentage decreased to 16.9% during mid L3 to L4, showing approximately 38% down-regulation, and a similar level of down-regulation (35.5%) was observed in line 2 (Figure 7Aa,b and C). In contrast, no down-regulation was observed in the control line carrying the integrated transgene *qIs56[P_lag-2_::gfp]*, in which *gfp* was expressed under the control of the *P_lag-2_* promoter (Figure 7Ba,b and C). These results show that BLMP-1 levels drop significantly when entering the mid L3 stage. Although the difference may be due to differences in sensitivity of the *gfp* reporters, given the previous result that *blmp-1* transcriptional repression was detected in late, but not mid, L3 stage, this suggests that BLMP-1 degradation occurs approximately 3 hours earlier than transcriptional repression.

Next, we examined whether *dre-1* was required for this decrease in GFP::BLMP-1 fusion protein levels. In two independent transgenic lines of the *dre-1* mutant, the percentage of worms with DTCs expressing GFP::BLMP-1 during the early L3 stage was similar to that seen during the mid L3-to-L4 stage (Figure 7Ac,d, and C), showing that *dre-1* is required for GFP::BLMP-1 degradation. Since loss of *dre-1* completely blocked the decrease in GFP::BLMP-1 levels, it is unlikely that any other gene acts in a redundant fashion with *dre-1* to regulate BLMP-1 stability.

### DRE-1 and BLMP-1 physically interact, as do their mammalian orthologs FBXO11 and PRDI-BF1

**Figure 8:**
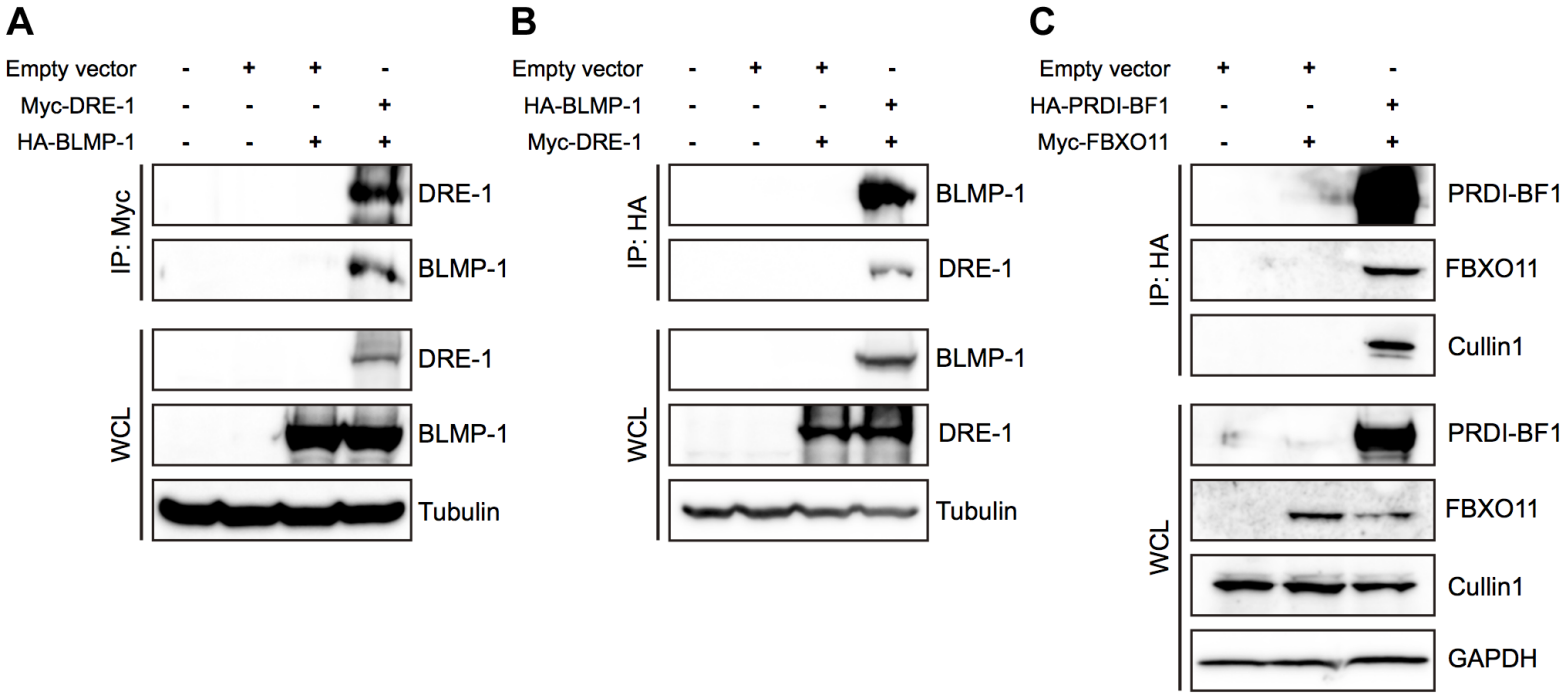
BLMP-1 and DRE-1 are co-immunoprecipitated in human cell cultures, as are their human orthologs PRDI-BF1 and FBXO11. HEK293T cells were transfected with the indicated plasmids. Expression of the indicated plasmids in the whole cell lysate (WCL) is shown in the bottom panels, with tubulin or GAPDH as the loading control. (A, B) Co-immunoprecipitation of *C. elegans* Myc-DRE-1 and HA-BLMP-1. (A) Myc-DRE-1 was immunoprecipitated with anti-Myc antibodies and Western blots were probed with anti-HA antibodies to detect HA-BLMP-1 and with anti-Myc antibodies to detect Myc-DRE-1. (B) HA-BLMP-1 was immunoprecipitated with anti-HA antibodies and Western blots were probed with anti-Myc antibodies to detect Myc-DRE-1 and with anti-HA antibodies to detect HA-BLMP-1. (C) Co-immunoprecipitation of *C. elegans* Myc-DRE-1 and HA-BLMP-1. HA-PRDI-BF1 was immunoprecipitated with anti-HA antibodies and Western blots were probed with anti-Myc antibodies to detect Myc-FBXO11 and with anti-cullin 1 antibodies to detect cullin 1.

The F-box protein of an SCF ligase complex binds to substrates and targets them for ubiquitin-mediated proteolysis [30]. To test the idea that BLMP-1 might be the target of DRE-1 in the SCF^DRE-1^ ligase complex, we examined whether DRE-1 and BLMP-1 physically interacted by co-immunoprecipitation in mammalian cell cultures. When Myc-tagged *dre-1* and HA-tagged *blmp-1* were transfected into HEK293T cells, then lysates were subjected to immunoprecipitation with anti-Myc antibody and Western blotting with anti-HA antibody, BLMP-1 was co-immunoprecipitated with DRE-1, while no signal was seen in the singly transfected controls (Figure 8A). The reciprocal experiment using immunoprecipitation with anti-HA antibody and Western blotting with anti-Myc antibody showed that DRE-1 was co-immunoprecipitated with BLMP-1 (Figure 8B). These results demonstrate that DRE-1 binds to BLMP-1 in HEK293T cells.

We next examined whether FBXO11 and PRDI-BF1, the respective human orthologs of DRE-1 and BLMP-1, also associate in HEK293T cells and found that Myc-tagged FBXO11 was co-immunoprecipitated with HA-tagged PRDI-BF1 in lysate of cells co-transfected with both, but not in lysate of cells transfected with only Myc-tagged FBXO11 (Figure 8C). This result shows association of FBXO11 and PRDI-BF1 in human cell cultures. In addition, HA-tagged PRDI-BF1 also pulled down endogenous CUL1 in co-immunoprecipitation experiments in HEK293T cells (Figure 8C), demonstrating the association of FBXO11, BLMP-1, and CUL1 in an SCF complex and suggesting that FBXO11 may regulate PRDI-BF1 stability through ubiquitin-mediated proteolysis. These results demonstrate a conserved interaction between DRE-1/FBXO11 and BLMP-1/PRDI-BF1 in both humans and *C. elegans*. The regulation of BLMP-1/PRDI-BF1 stability by DRE-1/FBXO11 in an SCF ubiquitin ligase complex may also be conserved in evolution.

## Discussion

### *blmp-1* acts in a heterochronic pathway to regulate the timing of the DTC dorsal turn

In this study, we identified a conserved transcription factor, BLMP-1, as an essential component of the heterochronic hierarchy during gonad development, and provided evidence that BLMP-1 levels are critical for the timing of DTC dorsal migration during gonadogenesis. BLMP-1 was present in DTCs only before the dorsal turn and disappeared when the DTC was about to make the dorsal turn. Loss of *blmp-1* resulted in precocious *unc-5* transcription and DTC dorsal migration, whereas constitutive expression of *blmp-1* delayed both events. These data show that *blmp-1* controls the temporal identity of DTCs by preventing them from undergoing a precocious dorsal turn. In addition, our results provide a molecular mechanism by which *daf-12*, *dre-1*, and *lin-29* promote the correct timing of DTC dorsal turn by timely down-regulation of *blmp-1*. In this model, *lin-29* and *daf-12* repress *blmp-1* transcription, which abolishes the synthesis of *blmp-1* mRNA, while *dre-1* mediates BLMP-1 degradation. Together, these two negative regulatory systems result in the efficient elimination of *blmp-1* activity, and thus alleviate the repression of the DTC dorsal turn.

### *daf-12*, *dre-1*, *a*nd *lin-29* function in parallel to reduce BLMP-1 levels prior to the DTC dorsal turn

Interestingly, *daf-12*, *dre-1*, and *lin-29* appeared to contribute to BLMP-1 down-regulation to different extents. For example, in the *dre-1*; *lin-29*, *dre-1; daf-12*, and *lin-29; daf-12* double mutants, 100%, 20%, or 5% of worms, respectively, showed persistent BLMP-1 expression beyond late L3 in the immunostaining experiment. This suggests that, of these three genes, loss of *dre-1* has the strongest effect on BLMP-1 down-regulation and loss of *daf-12* has the weakest effect. Using transcriptional and translational *gfp* reporters, it appeared that the DRE-1-dependent protein degradation process occurred at the mid L3 stage, slightly earlier than the transcriptional repression mediated by *lin-29* and *daf-12* (late L3 stage), although this difference could be due to different sensitivities of the *gfp* reporters. The notion that *lin-29* is more significant than *daf-12* in the repression of *blmp-1* was further supported by the observation that, using the *P_blmp-1_::dgfp* reporter, loss of *lin-29* increased *blmp-1* transcription in the late L3 to L4 stages, but loss of *daf-12* had no detectable effect on *blmp-1* expression (Figure 6C). Further experiments are necessary to test whether LIN-29 may inhibit *blmp-1* transcription by directly binding to the *blmp-1* genomic sequence.

It is intriguing that only 20% of the *dre-1; daf-12* double mutants had DTCs with detectable persistent BLMP-1, whereas 98% of these double mutants showed the retarded phenotype (Table 1). It is possible that, in the mutant, BLMP-1 persists at a low level beyond the detection limit of our system, but sufficient to block *lin-29* transcription and therefore abolish the DTC dorsal turn in the absence of *daf-12*. On the other hand, the retarded DTC migration phenotype of the *lin-29; daf-12* double mutant may not be caused solely by persistent BLMP-1 at the undetectable low level, but also by the loss of both transcription activators *lin-29* and *daf-12*, which function in a redundant fashion in the transcriptional activation of *unc-5* (see below and Figure S5).

As shown in Figure 5 A, BLMP-1::GFP in worms carrying *P_blmp-1_::blmp-1::gfp* was present and persisted beyond late L3 stage and the transgenic worms frequently showed a retarded DTC migration defect. A similar result was observed in worms carrying *P_lag-2_::blmp-1::gfp* (Huang and Wu, unpublished results). Interestingly, wild-type worms carrying *P_lag-2_::blmp-1* had normal DTC migration (Figure 1G), suggesting that BLMP-1 in the transgenic worms was properly degraded beyond L3 stage. These data and the observation that GFP::BLMP-1 expressed from the transgene *P_lag-2_::gfp::blmp-1* was degraded suggest that GFP tag at N-terminus or C-terminus make a difference in GFP fusion BLMP-1 degradation.

### Regulation of *unc-5* transcription

Using ChIP-seq, the modENCODE Consortium showed that BLMP-1 binds to the upstream sequence of *unc-5 in vivo* [26], suggesting that it might inhibit *unc-5* expression by binding to the regulatory region of the gene. In addition, using the *P_unc-5(4.6 kb)_::gfp* transgene, we found that inactivation of either *daf-12* or *lin-29* slightly reduced *unc-5* transcription, while loss of both genes completely abolished it (Figure S5). These results are consistent with the notion that the transcription factors DAF-12 and LIN-29 act together in a redundant fashion to activate *unc-5* transcription and promote the DTC dorsal turn. The consensus DAF-12 binding sequence can also be identified in the *unc-5* promoter (T.F. Huang and Y.C. Wu, unpublished results), raising the possibility that DAF-12 may directly bind to *unc-5* for its transcriptional activation.

### A double-negative feedback loop involving *blmp-1* and *lin-29* contributes to the DTC dorsal turn

**Figure 9:**
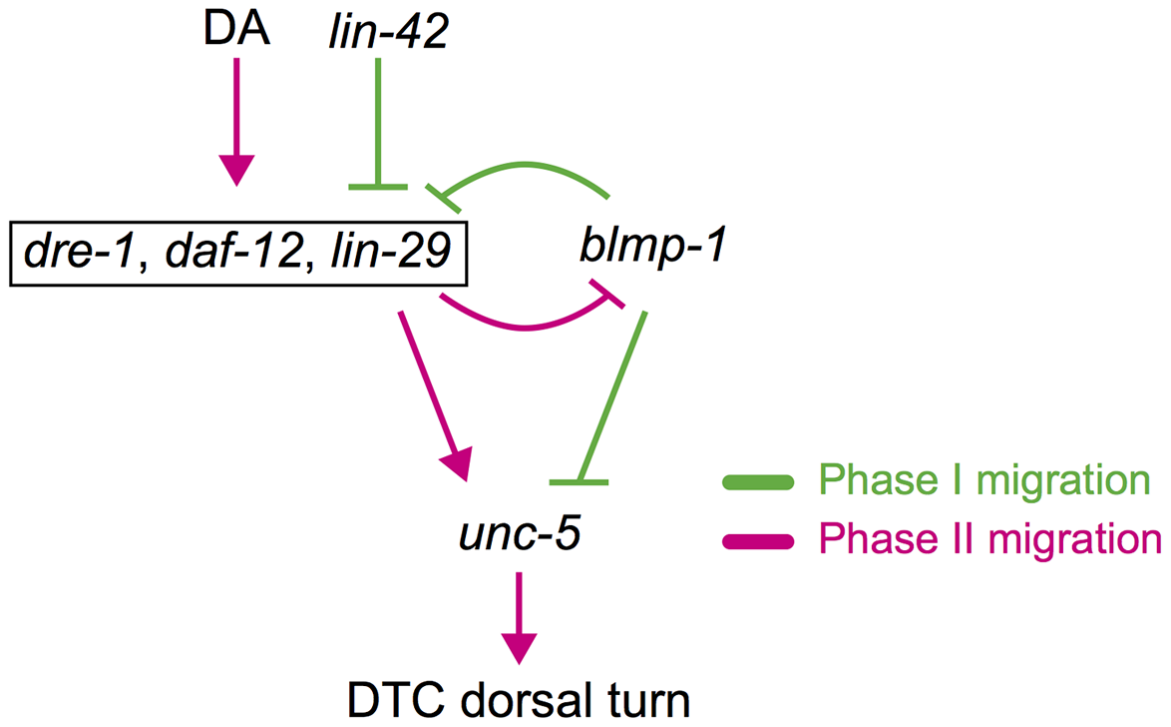
Spatiotemporal regulation of DTC migration. *blmp-1* and lin-42 negatively regulate the timing of the DTC dorsal turn, whereas *daf-12*, *dre-1*, and *lin-29* function in a redundant fashion to positively regulate the turn. Although *daf-12* is activated by DAs (dafachronic acids) and *lin-42* inhibits *daf-12* and *lin-29*, they are boxed together for convenience. A double-negative feedback loop between *blmp-1* and *lin-29* may contribute to the switch-like turning process of DTCs (see text for detail). Gene interactions that occur in phase I migration are shown in green and those that occur in phase II migration are shown in red.

The DTC dorsal turn can be characterized by a specific transition in which the DTC moves irreversibly from phase I centrifugal migration into phase II dorsal migration. Phase I centrifugal migration occurs in the “*blmp-1*-on” state (high BLMP-1 and low UNC-5 levels) and phase II dorsal migration occurs in the“*blmp-1*-off” state (low BLMP-1 and high UNC-5 levels). A double-negative feedback loop, in which two genes mutually repress each other directly or indirectly, is commonly utilized to generate switch-like bistable responses during the progression of cellular and developmental processes [31]. Our data suggest that *lin-29* and *blmp-1* act in a double negative feedback loop that helps maintain DTCs in one of the bistable “*blmp-1*-on” and “*blmp-1*-off” states. On the basis of our results, we propose a molecular model for the switch-like process of the DTC dorsal turn. In L2 and early L3, BLMP-1 is expressed and represses *lin-29* transcription (Figure 9). The double-negative feedback loop keeps DTCs in the “*blmp-1*-on” state, thus repressing DTC dorsal migration. In late L2, the hormones known as dafachronic acids (DA) are generated from dietary cholesterol and bind to the ligand binding domain of DAF-12 [32]. The DAF-12-DA complex promotes the L2-to-L3 transition [32], but is insufficient to repress *blmp-1* transcription. During early to mid L3, *dre-1* expression is initiated [4], which reduces BLMP-1 levels, probably through ubiquitin-mediated proteolysis. The decrease in BLMP-1 levels shifts the steady state toward low BLMP-1 levels and high LIN-29 levels (*blmp-1 off*). In late L3, accumulation of LIN-29 locks the DTCs in the “*blmp-1*-off” state through the negative feedback loop, thus allowing DTCs to switch to dorsal migration. This gene regulatory circuit integrates the temporal and spatial signals and coordinates with overall development of the organism to direct DTC dorsal migration during organogenesis.

Expression of *lin-29* is negatively regulated by *lin-42* [33]. LIN-42 is similar to the Period (Per) family of circadian rhythm proteins and functions as a member of the heterochronic pathway, regulating temporal cell identities [33], [34]. However, how *lin-42* regulates the expression of *lin-29* is not clear. *lin-42* also genetically interacts with *daf-12*. During development, when conditions are unfavorable, due to starvation, crowding or high temperature, *daf-12* promotes diapauses and formation of duaer larvae [5], [35], [36]. Previous studies show that *daf-12* represses expression of *lin-42* during dauer formation [37] and that *lin-42* antagonizes the dauer-inducing signal from ligand-free DAF-12 [38]. However, whether *daf-12* and *lin-42* also regulate each other in the timing of DTC dorsalward turn during gonadaogenesis needs to be examined in the future.

### *blmp-1* is important for DTC migration along the A-P axis

In addition to ventral-to-dorsal migration, *blmp-1* affects DTC migration along the AP axis. As shown in Figure 1D, some DTCs migrated obliquely with respect to the dorsal-ventral axis in *blmp-1* mutants, suggesting simultaneous execution of centrifugal phase I and dorsal phase II movement. This result implies that the mutual exclusion of dorsalward and centrifugal migration in wild type animals was impaired in the *blmp-1* mutants. Moreover, as shown in Figure 1E and 1F and Table S1, the reversal of migration direction was frequently observed in the centripetal phase III migration of *blmp-1* mutants. Expression of *unc-5* by the *lag-2* promoter from transgene *P_lag-2_::unc-5* resulted in DTC migration defects, including the reversal of the migration direction during the centripetal phase III migration (Huang, T.F. and Wu, Y.C., unpublished results), similar to those seen in *blmp-1* mutants (Table S1). Further experiments are necessary to test whether the phase III DTC migration defect of *blmp-1* mutants may be caused by an accumulative high level of *unc-5* in the DTCs.

### *blmp-1* is essential for multiple cellular and developmental processes

Previous studies have shown that *blmp-1* mutants have a dumpy phenotype, a weak cuticle sensitive to oxidative stress, and defective adult alae, showing that *blmp-1* is important for normal body length, oxidative stress resistance, and alae formation [39]. In addition, *blmp-1* is important for pharynx and [40] male tail morphogenesis [41]. Under Nomarski optics, we confirmed the *blmp-1* alae defect, as 40% of *blmp-1(s71)* mutants had incomplete alae and the rest had no alae in the adult stage (Figure S3C). The alae are continuous ridged cuticular structures synthesized by the lateral epidermal seam cells at the larval to adult (L/A) transition and serve as a specific marker of adult fate. The seam cells undergo asymmetric cell divisions at each larval stage and exit the cell cycle at the L/A transition. The *blmp-1(s71)* mutant was found to have a normal number of seam cells at the late L4 and early adult stage (Figure S3D), indicating that seam cell division was normal. These results and the abnormal alae phenotype suggest that *blmp-1* is essential for the adult, but not the larval, fate of the epidermal seam cells.

The *dre-1* mutant shows precocious formation of adult alae [4]. We therefore tested the genetic interaction between *dre-1* and *blmp-1* in the seam cell heterochronic hierarchy. About 8% of *dre-1(dh99)* mutants had adult alae at the early L4 stage (Figure S3D), confirming a role of *dre-1* in preventing the adult fate of the seam cell [4]. At the adult stage, 35% of *dre-1(dh99)* mutants had incomplete alae (Figure S3Cc and D). Interestingly, the *blmp-1; dre-1* double mutant had a weaker defect in alae formation than the *blmp-1* single mutant, as 23% of double mutants and 60% of *blmp-1* single mutants had no alae (Figure S3C and D). This result shows that *dre-1* partially suppresses the *blmp-1* alae abnormality and, therefore, *blmp-1* may genetically act upstream of, or in parallel to, *dre-1* in the specification of the adult seam cell fate, at least in terms of alae formation.

*blmp-1*(*tm548* or *s71*) mutants have a weak uncoordinated movement phenotype [39]. Although *blmp-1* expression has been previously observed in neurons using reporter transgene assays [26], we failed to detect BLMP-1 in neurons. It is possible that our anti-BLMP-1antibody system was not sensitive enough to detect a low amount of protein in neurons. Alternatively, the neuronal expression of *blmp-1* revealed by the transgene may be ectopic and not reflect the endogenous expression pattern.

Like the *blmp-1* mutations, *lin-42* RNAi causes both a precocious DTC dorsal turn [33] and precocious *lin-29* expression in L2, one stage earlier than in the wild-type [33]. However, the *lin-42(RNAi)* and *blmp-1* mutations induce precocious dorsal migration at different larval stages, i.e. in L2 for *lin-42(RNAi)* [33] and early L3 in the case of *blmp-1* (Figure 3). Similar to loss of *blmp-1*, precocious expression of *lin-29* under the control of the *lag-2* promoter caused the DTCs to undergo a precocious dorsal turn in early L3, but not L2. These results suggest that *lin-42* regulates most, if not all, of the genes necessary for the DTC dorsal turn, while *blmp-1* regulates the temporal expression of only a subset, including *lin-29*.

### The conserved interactions between BLMP-1/PRDI-BF1 and DRE-1/FBXO11

DRE-1 has two human orthologs FBXO10 and FBXO11, which are localized in the cytoplasm and nucleus, respectively [29]. FBXO10 and DRE-1 mediate, respectively, the degradation of anti-apoptotic protein Bcl2 or CED-9 to promote the death of a cell [29]. Recently, FBXO 11 has been shown to target the pro-oncogene product BCL6 for degradation and to be inactivated in diffuse large B cell lymphomas [42]. BCL6, a zinc finger transcription factor, regulates the transcription of a variety of genes involved in B cell development, differentiation, and activation [43], [44] and is overexpressed in the majority of patients with aggressive diffuse large B cell lymphoma [45]. Using the Clustal Omega multiple sequence alignment website http://www.ebi.ac.uk/Tools/msa/clustalo/, we found that Bcl6 shares sequence similarity with BLMP-1 (20%) and PRDI-BF1 (24%). These observations and our result showing that DRE-1/FBXO11 and BLMP-1/PRDI-BF1 are associated in human cell cultures indicate that DRE-1, FBXO10 and FBXO11 target different proteins for degradation in different cellular or developmental contexts, i.e. DRE-1 targets BLMP-1 and CED-9, FBXO10 targets Bcl2 and FBXO11 targets PRDF-BF1 and Bcl6. It will be interesting to determine how target specification is regulated.

## Materials and Methods

### Strains

*C. elegans* strains were cultured at 20°C on NGM agar inoculated with *E. coli* OP50, as described previously [46]. The N2 Bristol strain was used as the reference wild-type strain. The mutations used were as follows: LGI, *blmp-1(s71, tk41, tm548, tp5), lin-29(n546)*; LGIII, *unc-119(ed3)*; LGV, *dre-1(dh99)*; LGX *daf-12(rh61rh411)*. The *blmp-1(tm548)* mutant was generated and provided by the National Bioresource Project in Japan. Strains CB4856 and RW7000 were used for single-nucleotide polymorphism (SNP) mapping [15], [47]. Strain OS1841 carrying the transgene P*_lin-29_*::GFP was kindly provided by S. Shaham [48].

### Genetics

To test whether the *tk41*, *tm548*, *s71* or *tp5* mutation is recessive or dominant, homozygous mutant hermaphrodites were crossed to wild-type males carrying the integrated *sur-5::gfp* transgene. The cross-progeny hermaphrodites carrying the *sur-5::gfp* transgene were scored for the dumpy (Dpy) and DTC migration phenotypes using Nomarski microscopy. In these crosses, all heterozygous cross-progeny hermaphrodites were wild-type, showing that these mutations are recessive.

In complementation tests, the *tk41*, *tm548* or *tp5* homozygous hermaphrodites were crossed to *s71* males carrying the *sur-5::gfp* transgene, the cross-progeny hermaphrodites with the transgene were scored for the Dpy and DTC migration phenotypes using Nomarski microscopy. In these crosses, all of the mutations tested failed to complement *s71*.

We positioned *blmp-1(s71)* on the basis of the Dpy phenotype within the region between snpF45H11 and snpY106g6h, which correspond to cosmids F45H11 and F37D6, respectively, using three-factor mapping with *unc-40* and *unc-75* and subsequent SNP mapping as previously described [47]. The following cross demonstrates that *blmp-1* is located between *unc-40* and snpY106g6h: from a *blmp-1(s71)unc-75*/++ (CB4856) hermaphrodite 37/37 Dpy non-Unc recombinant progeny segregated neither snpY106g6h nor snpF59C6, and 6/6 nonDpy Unc recombinant progeny segregated snpY106g6h and snpF59C6. From an *unc-40 blmp-1 (s71)*/++(CB4856) hermaphrodite 85/91 nonDpy Unc recombinant progeny segregated snpY106g6h and snpF59C6, 1/91 recombinant progeny segregated snpY106g6h and 5/91 recombinant progeny segregated neither snpY106g6h nor snpF59C6. The following cross demonstrates that *blmp-1* is located right of snpF45H11: from an *unc-40 blmp-1(s71)*/CB4856 hermaphrodite 3/171 Dpy nonUnc recombinant progeny segregated snpF45H11.

### Genetic screen

We performed genetic screening for mutants defective in DTC migration under a dissecting microscope, as described in Nishiwaki, 1999, and isolated the mutation *tk41*. In an independent screen for mutants with a DTC migration defect using Nomarski microscopy, we isolated the mutation *tp5*.

### Analysis of the DTC migration phenotype

The DTC migratory patterns of the wild-type and mutants were determined by observing the shape of the gonadal arms in the adult stage. Worms were mounted on a 4% agar pad containing 20 mM NaN_3_ and observed on a microscope with Nomarski optics. For the time course analysis of DTC migration, wild-type and *blmp-1(s71)* mutants at the indicated time points were collected and scored under the Nomarski microscope.

### DNA construct

To obtain the 5′ end of *blmp-1* cDNA, the first three exons were amplified by RT-PCR using a forward primer corresponding to the SL1 sequence and the reverse primer *blmp-1*_exon_3/r (see Table S5 for detailed information). The PCR product was fused with the *blmp-1* cDNA fragment from the yk487b7 clone by fusion PCR [49], and the resulting product was cloned into the vector pGEM-T Easy (Promega) to generate pYW687, containing the full-length *blmp-1* cDNA.

To construct *P_lag-2_::blmp-1* (pYW798), *blmp-1* cDNA was PCR-amplified from pYW687 using primers DPY-24-KpnI/f and DPY-24-KpnI/r and the product inserted into pPD49_26/P*_lag-2_* via the *Kpn*I site.

Two *unc-5* transcriptional *gfp* constructs were generated. The *P_unc-5(4.6 kb)_* DNA fragment was PCR-amplified from *C. elegans* genomic DNA using primers *P_unc-5_*_4.6 kb/f and *P_unc-5_*_4.6 kb/r and inserted into the vector pGEM-T Easy. The *P_unc-5(4.6 kb)_* fragment of the resulting construct was then inserted into the vector pPD95.77 (A. Fire) *via Sph*I/*Sal*I sites to generate *P_unc-5(4.6 kb)_::gfp*. A similar approach was used to generate *P_unc-5(1 kb)_::gfp*. The *P_unc-5(1 kb)_* fragment was PCR-amplified from genomic DNA using primers *P_unc-5_*_1 kb/f and *P_unc-5_*_1 kb/r and cloned into the vector pGEM-T Easy, then the *P_unc-5(1 kb)_* fragment was excised from the resulting plasmid and inserted into the vector pPD95.75 (A. Fire) via the *Xma*I site. To generate *P_unc-5(1 kb)_::mCherry*, the *gfp* fragment of the plasmid *P_unc-5(1 kb)_::gfp* was replaced by *mCherry* cDNA.

The *P_unc-5(1 kb)_* fragment was expected to contain the regulatory region for proper *unc-5* function in guiding DTC migration, as the *P_unc-5(1 kb)_::unc-5::gfp* plasmid is sufficient to rescue the DTC migration defect of the *unc-5* mutant (our unpublished data). The *P_unc-5(1 kb)_::unc-5::gfp* plasmid was generated by fusion PCR by fusing two PCR-amplified products corresponding to *P_unc-5(1 kb)_::unc-5(cDNA)* and *gfp*::*unc-54* 3′ UTR from plasmid pU5HA (J. Culotti) or pPD95.75, respectively.

The *P_blmp-1_::dgfp* fragment was generated by fusion PCR by fusing two PCR products corresponding to *P_blmp-1_* and the region containing *dgfp* and the *unc-54* 3′ UTR. *P_blmp-1_* was PCR-amplified from genomic DNA using primers D24-5end5kb and d24Pgfp/r, and the region containing *dgfp* and *unc-54 3′UTR* was PCR-amplified from pPD95.75PEST (pYW807), which contains *dgfp*, using primers GFP/f and D. The two PCR products were then mixed and fused by fusion PCR using primers d24-5kbf-nest and D′. The *P_blmp-1_::blmp-1::gfp* fragment was generated by fusion PCR by fusing two PCR products corresponding to *blmp-1* genomic DNA and the region containing *gfp* and *unc-54* 3′ UTR from the pPD95.75 plasmid [50]. *blmp-1* genomic DNA was PCR-amplified from wild-type genomic DNA using primers D24-5end5kb and d24gfp/r, and the region containing *gfp* and the *unc-54 3′* UTR was PCR-amplified from the pPD95.75 plasmid using primers GFP/f and D, then the two PCR products were fused by fusion PCR using primers d24-5kbf-nest and D′.

The primers used in this work are listed in Table S6.

### Transgenic worms

Transgenic worms were generated by microinjection of the indicated plasmid, as described previously [51]. For genetic rescue experiments, cosmids and plasmids were microinjected into the indicated strains and the indicated phenotype of the stably transmitting lines scored using DIC optics. P*_blmp-1_::blmp-1::gfp* (20 ng/µl) was co-injected with the *unc-119* rescuing plasmid (100 ng/µl) [52] into the *unc-119(ed3)* mutant to generate *tpEx49* transgenic worms. For the *unc-5* transcription assay, *P_unc-5(4.6 kb)_::gfp*, *P_unc-5(1 kb)_::gfp*, or *P_unc-5(1 kb)_::mCherry* (20 ng/µl) was co-injected with the marker *P_myo-2_::gfp* (2 ng/µl), which expresses *gfp* in the pharynx [26], [53], as described previously [51]. To determine how *blmp-1* was down-regulated during larval development, the indicated plasmids (20 ng/µl) were co-injected with *P_myo-2_::gfp* (2 ng/µl) into wild-type worms to generate transgenic worms, and the resulting transgenes were crossed to the indicated heterochronic mutants. For the *blmp-1* cell autonomy assay, *P_lag-2_::blmp-1* or *P_blmp-1_::blmp-1* (20 ng/µl) was co-injected with *P_myo-2_::gfp* (2 ng/µl) into wild-type worms to generate worms carrying the respective transgenes, then the *blmp-1(s71)* mutation was introduced into these transgenic worms by crossing to generate the *blmp-1(s71)* mutant carrying either the *P_lag-2_::blmp-1* or *P_blmp-1_::blmp-1* transgene.

The transgenes used in this work were listed in Table S5.

### Antibodies and immunostaining

The *blmp-1* cDNA fragment from the plasmid pYW687 was digested with *EcoR*I and cloned into the vectors pGEX5X-1 (GE Healthcare) and pRSET B (Invitrogen) at their *EcoR* I sites to generate the respective constructs pYW802 and pYW691. The GST-BLMP-1 and HIS-BLMP-1 fusion proteins were present in inclusion bodies and were purified using standard methods [54]. Polyclonal rabbit antibodies against GST-BLMP-1 were generated and affinity-purified using 6His-tagged BLMP-1 as described previously [55] and the purified antibodies recognized GST-BLMP-1, but not GST, on a Western blot, indicating their BLMP-1 specificity. The immunostaining method was adopted from a previously published protocol [56], with slight modification. The animals were fixed for 1 h at 4°C in 80 mM KCl, 20 mM NaCl, 10 mM EGTA, 5 mM spermidine, 0.03 mM PIPES, 25% methanol, and 2% formaldehyde, then incubated overnight at 4°C with a 1∶100 dilution of the purified anti-BLMP-1 antibodies in PBS, 0.2% BSA, 0.5% Triton X-100 (PBT), then for 2 h at room temperature with rhodamine red-X (RRX)-conjugated donkey-anti-rabbit IgG antibodies (Jackson ImmunoResearch Laboratories; 1∶100 in PBT). For immunostaining of the *lin-29(RNAi);daf-12(rh61rh411)* mutants, the worms were mounted on a gelatin-chromic potassium sulfate-subbed slide and immunostained with anti-BLMP-1 antibodies, as described previously [57]. Worms were freeze-cracked and fixed in 95% ethanol for 10 min, then in 2% paraformaldehyde for 10 min, then incubated sequentially overnight at room temperature with purified anti-BLMP-1 antibodies (1∶1000 in PBT), followed by RRX-conjugated donkey-anti-rabbit IgG antibodies (Jackson ImmunoResearch Laboratories; 1∶1000 in PBT). They were then mounted with 2 µl of DABCO anti-bleaching reagent (Fluka) and 1 µl of DAPI (0.5 µg/ml) and observed using a Zeiss Axioplan 2 microscope equipped with a digital camera (AxioCam; Carl Zeiss, Inc.).

### RNA interference

To knock down *blmp-1*, *skr-1*, *cul-1*, *rbx-1*, *dre-1*, or *lin-29*, RNA interference (RNAi) was performed either by feeding worms with bacteria expressing the double stranded RNA for the indicated genes or by injecting the double stranded RNA for the indicated genes as described previously [58], [59]. To generate the *lin-29* RNAi construct, the region corresponding to exons 3–9 of *lin-29* was PCR-amplified from the yk1430g04 plasmid, which contains *lin-29* cDNA (Y. Kohara, Japan), and cloned into the L4440 vector. The *blmp-1*, *srk-1*, *cul-1*, and *rbx-1* RNAi constructs were obtained from the Ahringer RNAi library.

### HEK293T cell culture and drug treatments

HEK293T cells were grown in Dulbecco's modified Eagle's medium (Thermo Inc) containing 10% fetal bovine serum, 100 U/ml of penicillin, and 100 µg/ml of streptomycin (all from Life technologies) and were transfected with the indicated plasmids using Lipofectamine 2000 (Invitrogen). MG132 (20 µM) was added to the medium 2 h before the cells were harvested to block protein degradation by the proteasome.

### Co-immunoprecipitation and immunoblotting

Cells were harvested using pre-chilled PBS and lysed using 1% NP-40 lysis buffer with protease inhibitor (Roche). HA-tagged or Myc-tagged fusion proteins were immunoprecipitated by incubation overnight at 4°C with anti-HA or anti-Myc agarose beads (Sigma) and bound protein identified by Western blotting using antibodies against HA (Covance), Myc (BD Biosciences), or CUL1 (Zymed). Antibodies against tubulin (Sigma) or GAPDH (Abnova) were used on the loading controls.

## Supporting Information

Figure S1 Molecular Cloning of *blmp-1*. (A) The genetic map near the *dpy-24*/*blmp-1* locus on chromosome I is shown above. The cosmid clones shown in the middle and the open reading frames of the cosmid F25D7 shown below were tested for their abilities to rescue the *blmp-1(s71)* mutant phenotypes. The gene structure and transcription direction (indicated by an arrow) for each open reading frame is shown. (B) Rescue of the phenotype responsible for the DTC migration defect, dumpy phenotype or embryonic lethality of *blmp-1(s71)* mutants by germline transformation using genomic DNA clones. Plus sign, rescue; minus sign, no rescue. n.d., not determined.
(TIFF)

Figure S2 Sequence alignment of BLMP-1 and related proteins. Sequence alignment of BLMP-1, mouse Blimp-1, and human PRDI-BF1. The light gray and black shading indicates, respectively, residues that are identical in two or three proteins, while the dark gray shading with white letters indicate similar residues in two proteins. The PR domain is indicated by double underlining and the zinc fingers by circles, the predicted NLS is boxed by a rectangle, and the positions of the *blmp-1* mutant alleles are indicated by arrowheads.
(TIFF)

Figure S3 Loss of *blmp-1* results in partial penetrant embryonic lethality, a dumpy phenotype, and a defect in alae formation. (A) *blmp-1* mutants are dumpy. DIC images of wild-type and *blmp-1(s71)* worms 24 h after reaching adulthood. Scale bar, 100 µm. (B) *blmp-1* embryos have a partial penetrant embryonic lethality. The table shows the percentages of embryos that did not hatch after 24 h. The progeny derived from three worms was counted for each genotype. (C) DIC images of wild-type (a), *blmp-1(s71)* (b), *dre-1(dh99)* (c) and *blmp-1(s71); dre-1(dh99)* (d) adults. Alae are indicated by arrows. Scale bar, 10 µm. (D) The seam cell phenotype of the indicated mutants. The seam cell numbers and alae were scored at the indicated developmental stages. The seam cell-specific marker SCM::GFP allows visualization of seam cell nuclei and was used to assay seam cell number. The apical junction marker AJM-1::GFP was also present in the transgene.
(TIFF)

Figure S4 *P_unc-5(1 kb)_::gfp* is expressed in DTCs during, and after, the dorsal turn. DIC and GFP images at different migration phases of DTCs in worms carrying the transgene *P_unc-5(1 kb)_::gfp*. DTCs are indicated by arrows.
(TIFF)

Figure S5 DAF-12 and LIN-29 activate *unc-5* transcription in a redundant fashion. (A) DIC and GFP images in the late L3 stage of a wild-type worm (a), *lin-29(RNAi)* (b) worm, *daf-12(rh61rh411)* worm (c), or *daf-12(rh61rh411); lin-29(RNAi)* worm (d) carrying the *P_unc-5(4.6 kb)_::gfp* transgene. Scale bar 20 µm. The intensity of the GFP signal is also weaker in the *lin-29* or *daf-12* single mutants than in the wild-type worms. (B) Percentages of worms of the indicated genotype carrying the *P_unc-5(4.6 kb)_::gfp* transgene with posterior DTCs expressing GFP after late L3. At least 50 worms were scored.
(TIFF)

Table S1 DTC migration patterns of *blmp-1* mutants.
(TIFF)

Table S2 The speed of DTC migration is similar in wild-type and *blmp-1* mutants.
(TIFF)

Table S3 Genetic interactions of *blmp-1* and *unc-5*.
(TIFF)

Table S4 DRE-1 functions in an SCF complex to promote the DTC dorsal turn.
(TIFF)

Table S5 List of transgenic strains and transgenes used in this work.
(TIFF)

Table S6 Sequences of the primers used in the PCR reactions for cloning.
(TIFF)
