# Supplementary figures and images for: BLMP-1/Blimp-1 Regulates the Spatiotemporal Cell Migration Pattern in C. elegans
Source: PLoS Genet. 2014 Jun 26;10(6):e1004428. doi: 10.1371/journal.pgen.1004428 (PMC4072510; doi:10.1371/journal.pgen.1004428)

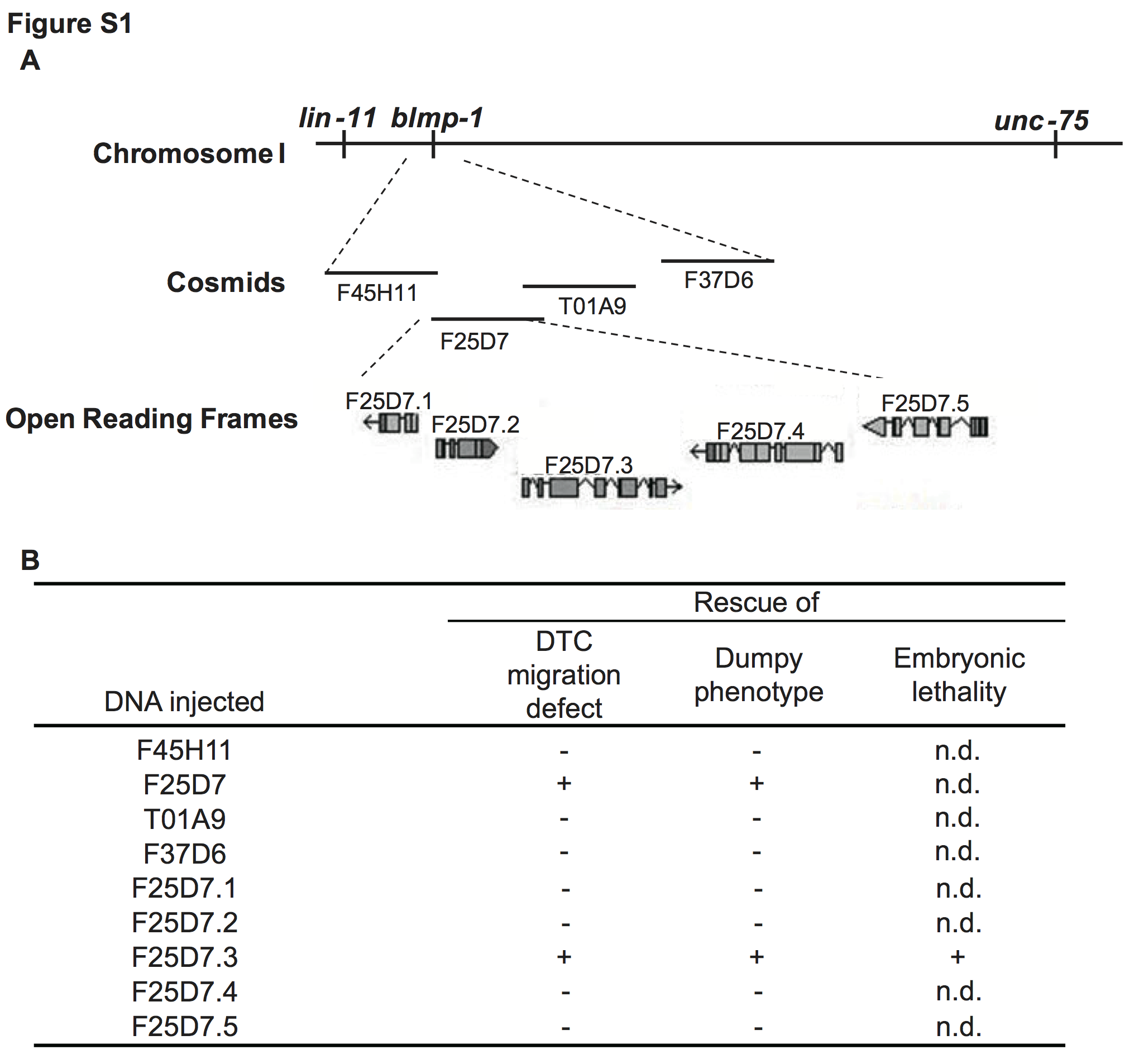

Supplement: Figure S1 — Molecular Cloning of blmp-1. (A) The genetic map near the dpy-24/blmp-1 locus on chromosome I is shown above. The cosmid clones shown in the middle and the open reading frames of the cosmid F25D7 shown below were tested for their abilities to rescue the blmp-1(s71) mutant phenotypes. The gene structure and transcription direction (indicated by an arrow) for each open reading frame is shown. (B) Rescue of the phenotype responsible for the DTC migration defect, dumpy phenotype or embryonic lethality of blmp-1(s71) mutants by germline transformation using genomic DNA clones. Plus sign, rescue; minus sign, no rescue. n.d., not determined. (TIFF) [file pgen.1004428.s001.tiff]

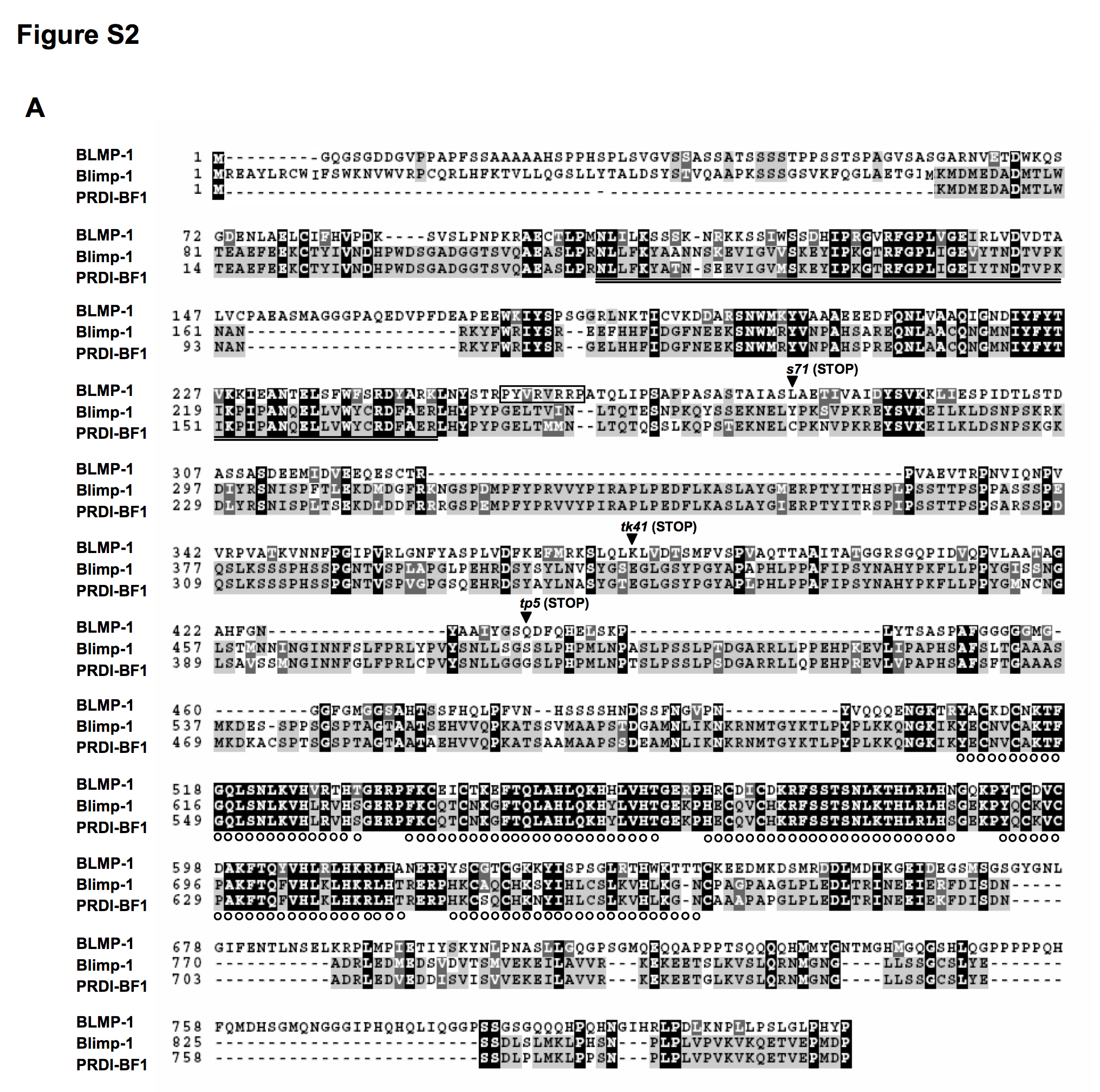

Supplement: Figure S2 — Sequence alignment of BLMP-1 and related proteins. Sequence alignment of BLMP-1, mouse Blimp-1, and human PRDI-BF1. The light gray and black shading indicates, respectively, residues that are identical in two or three proteins, while the dark gray shading with white letters indicate similar residues in two proteins. The PR domain is indicated by double underlining and the zinc fingers by circles, the predicted NLS is boxed by a rectangle, and the positions of the blmp-1 mutant alleles are indicated by arrowheads. (TIFF) [file pgen.1004428.s002.tiff]

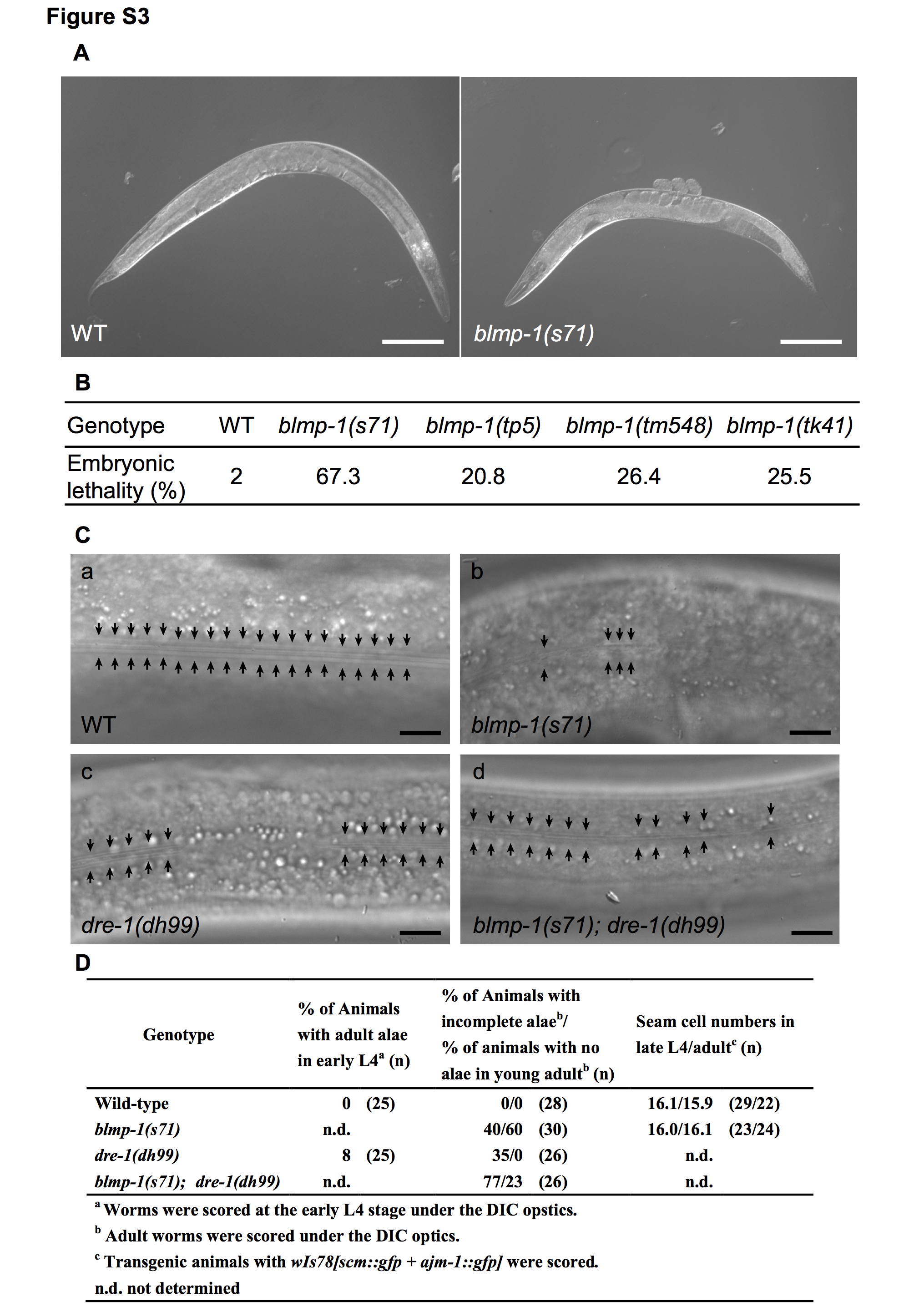

Supplement: Figure S3 — Loss of blmp-1 results in partial penetrant embryonic lethality, a dumpy phenotype, and a defect in alae formation. (A) blmp-1 mutants are dumpy. DIC images of wild-type and blmp-1(s71) worms 24 h after reaching adulthood. Scale bar, 100 µm. (B) blmp-1 embryos have a partial penetrant embryonic lethality. The table shows the percentages of embryos that did not hatch after 24 h. The progeny derived from three worms was counted for each genotype. (C) DIC images of wild-type (a), blmp-1(s71) (b), dre-1(dh99) (c) and blmp-1(s71); dre-1(dh99) (d) adults. Alae are indicated by arrows. Scale bar, 10 µm. (D) The seam cell phenotype of the indicated mutants. The seam cell numbers and alae were scored at the indicated developmental stages. The seam cell-specific marker SCM::GFP allows visualization of seam cell nuclei and was used to assay seam cell number. The apical junction marker AJM-1::GFP was also present in the transgene. (TIFF) [file pgen.1004428.s003.tiff]

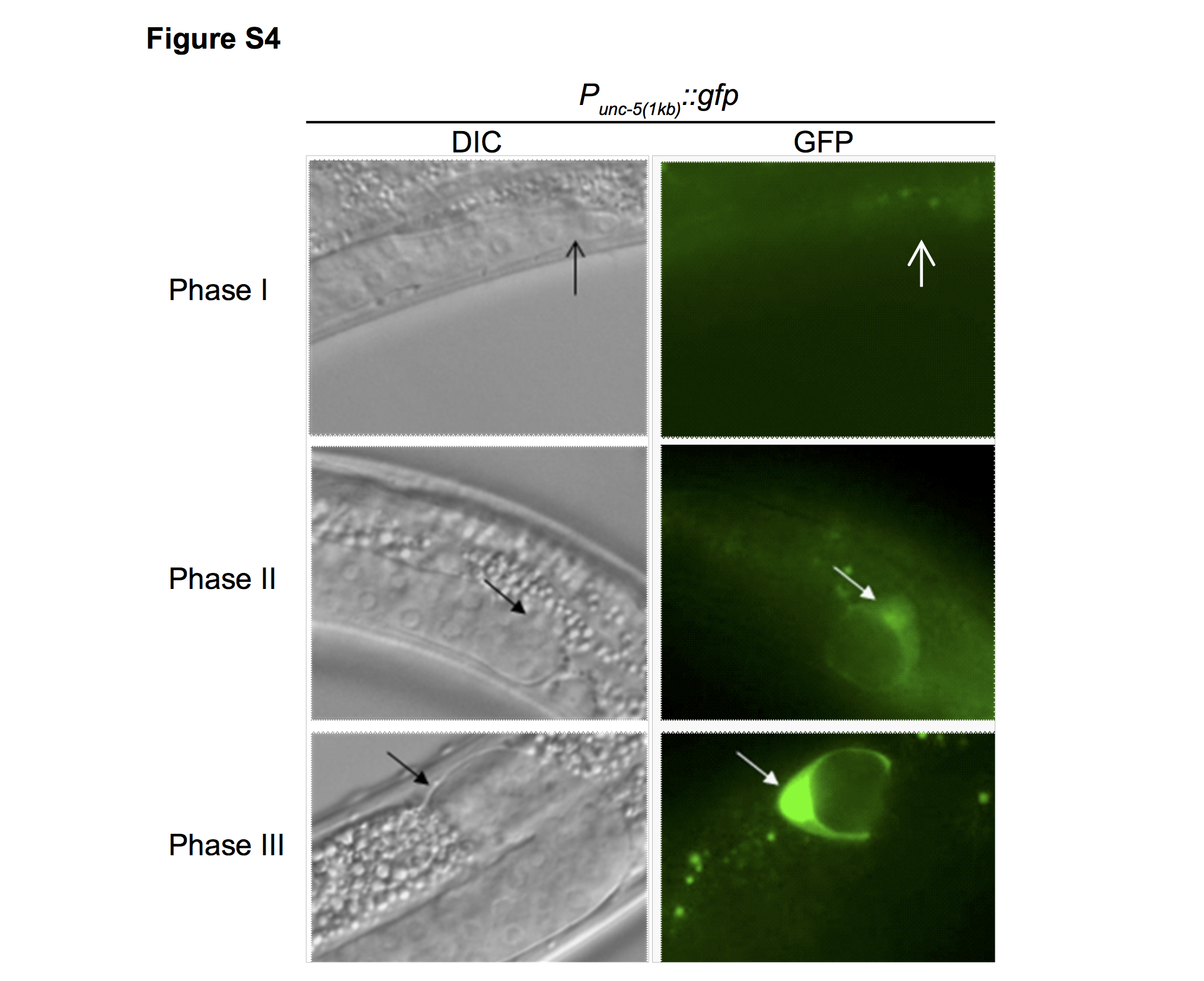

Supplement: Figure S4 — Punc-5(1 kb)::gfp is expressed in DTCs during, and after, the dorsal turn. DIC and GFP images at different migration phases of DTCs in worms carrying the transgene Punc-5(1 kb)::gfp. DTCs are indicated by arrows. (TIFF) [file pgen.1004428.s004.tiff]

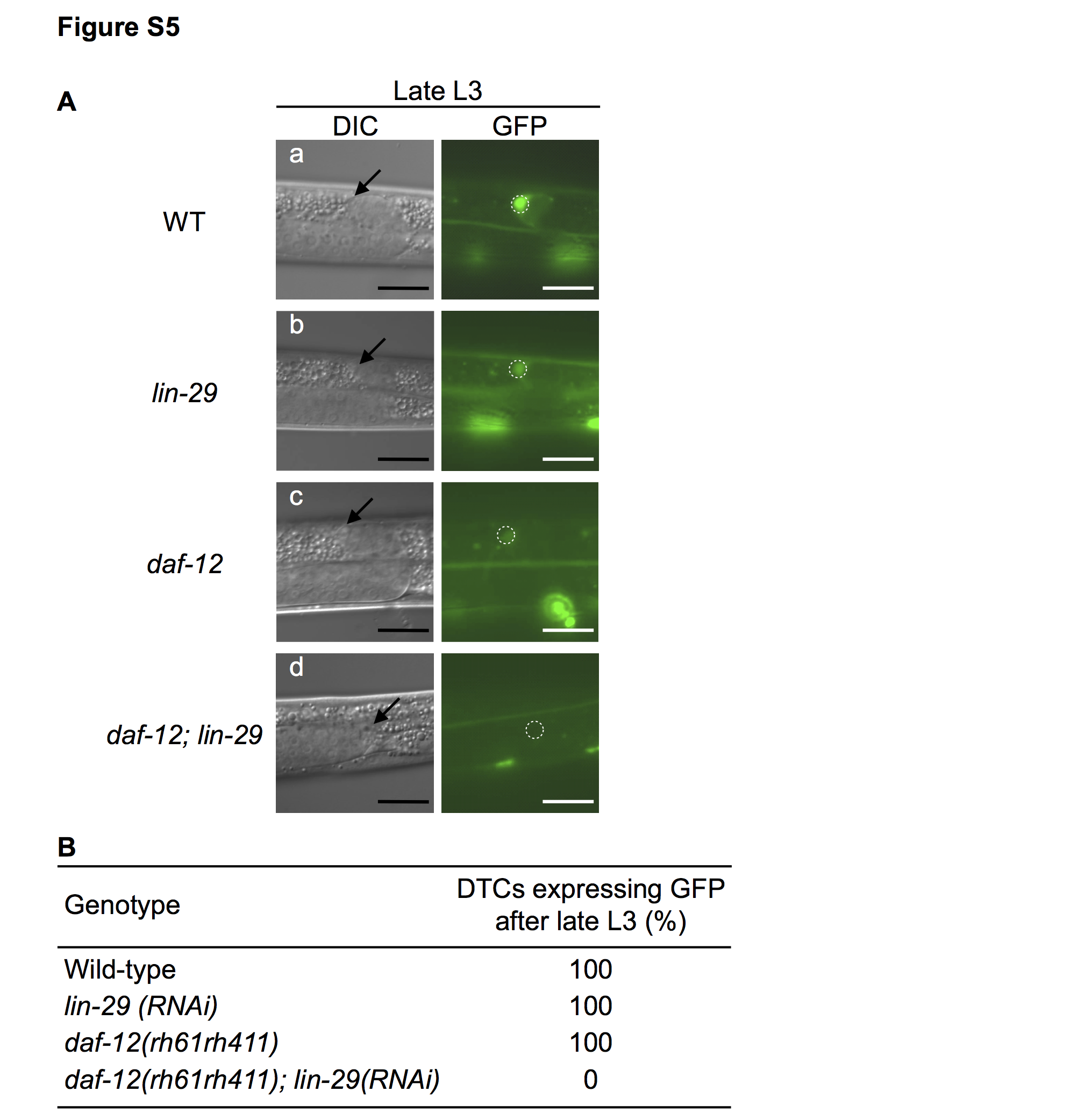

Supplement: Figure S5 — DAF-12 and LIN-29 activate unc-5 transcription in a redundant fashion. (A) DIC and GFP images in the late L3 stage of a wild-type worm (a), lin-29(RNAi) (b) worm, daf-12(rh61rh411) worm (c), or daf-12(rh61rh411); lin-29(RNAi) worm (d) carrying the Punc-5(4.6 kb)::gfp transgene. Scale bar 20 µm. The intensity of the GFP signal is also weaker in the lin-29 or daf-12 single mutants than in the wild-type worms. (B) Percentages of worms of the indicated genotype carrying the Punc-5(4.6 kb)::gfp transgene with posterior DTCs expressing GFP after late L3. At least 50 worms were scored. (TIFF) [file pgen.1004428.s005.tiff]

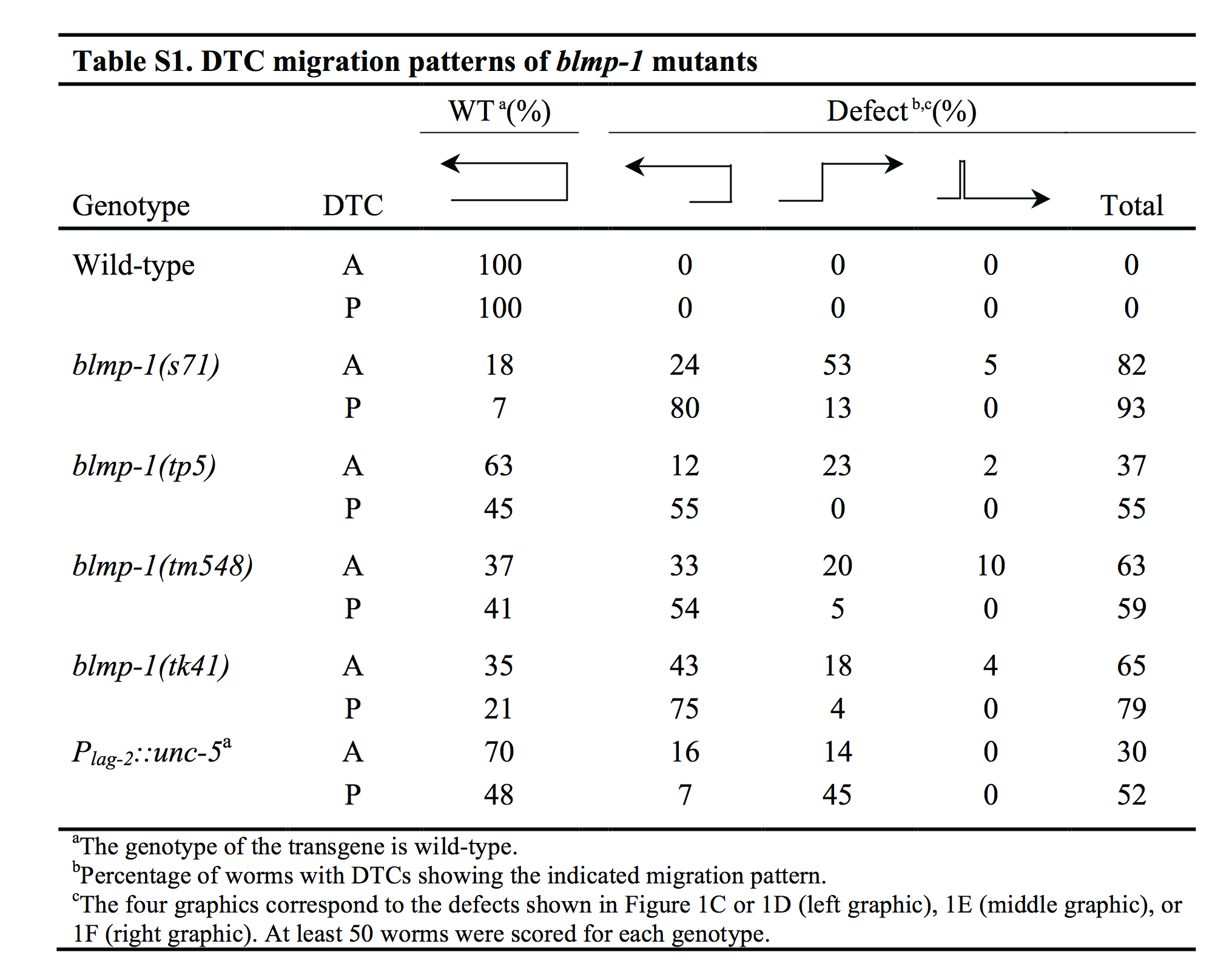

Supplement: Table S1 — DTC migration patterns of blmp-1 mutants. (TIFF) [file pgen.1004428.s006.tiff]

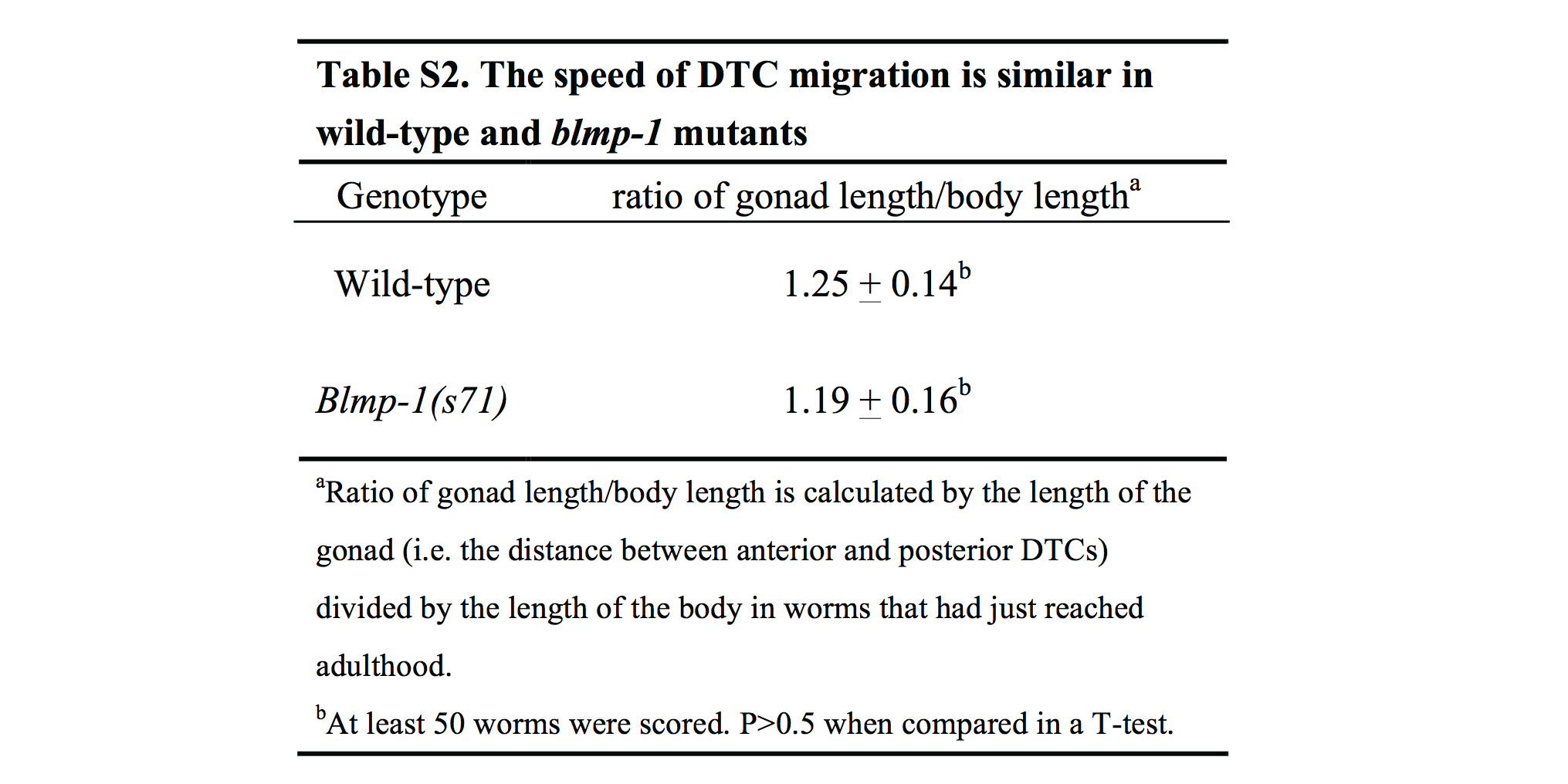

Supplement: Table S2 — The speed of DTC migration is similar in wild-type and blmp-1 mutants. (TIFF) [file pgen.1004428.s007.tiff]

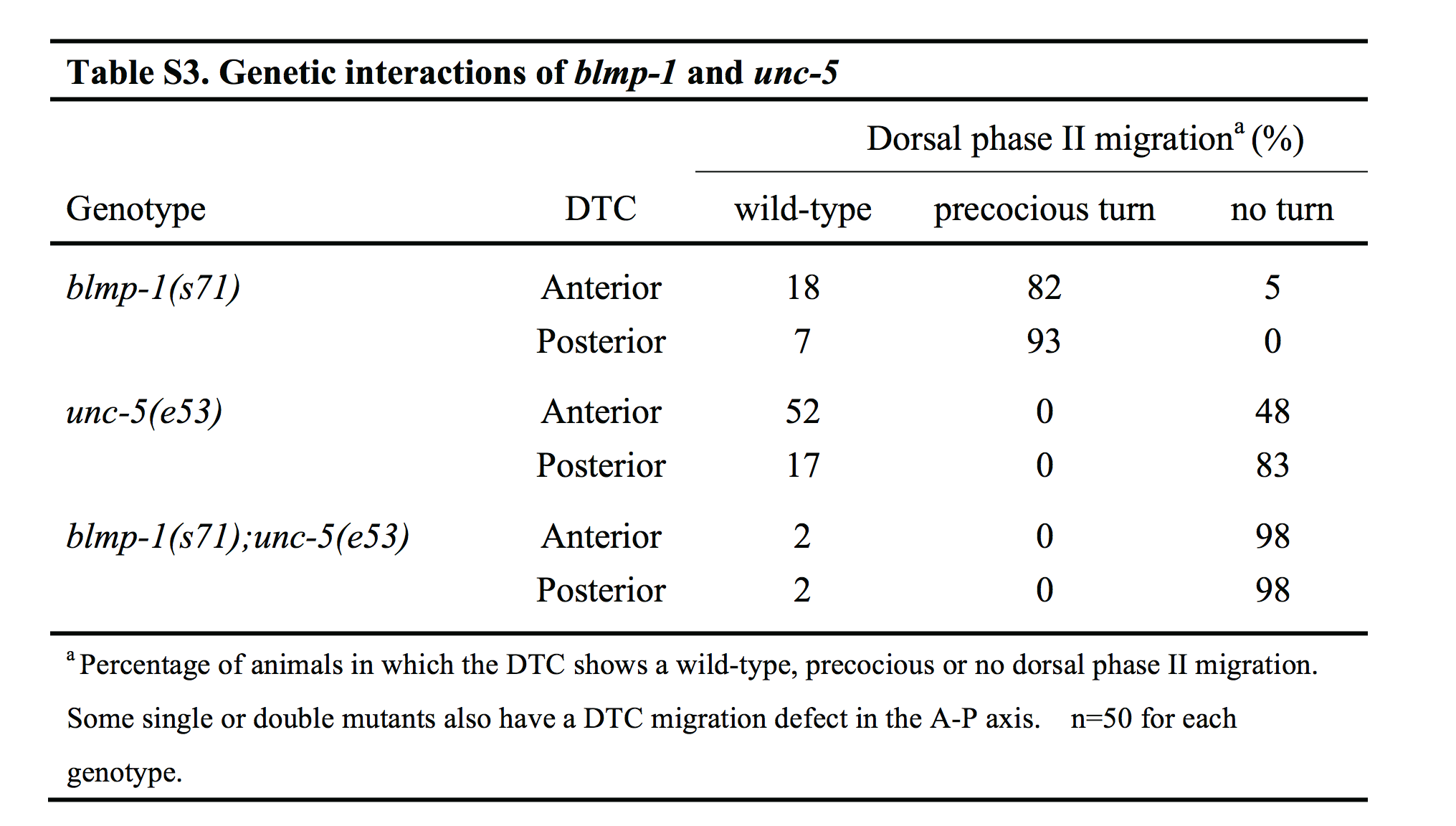

Supplement: Table S3 — Genetic interactions of blmp-1 and unc-5. (TIFF) [file pgen.1004428.s008.tiff]

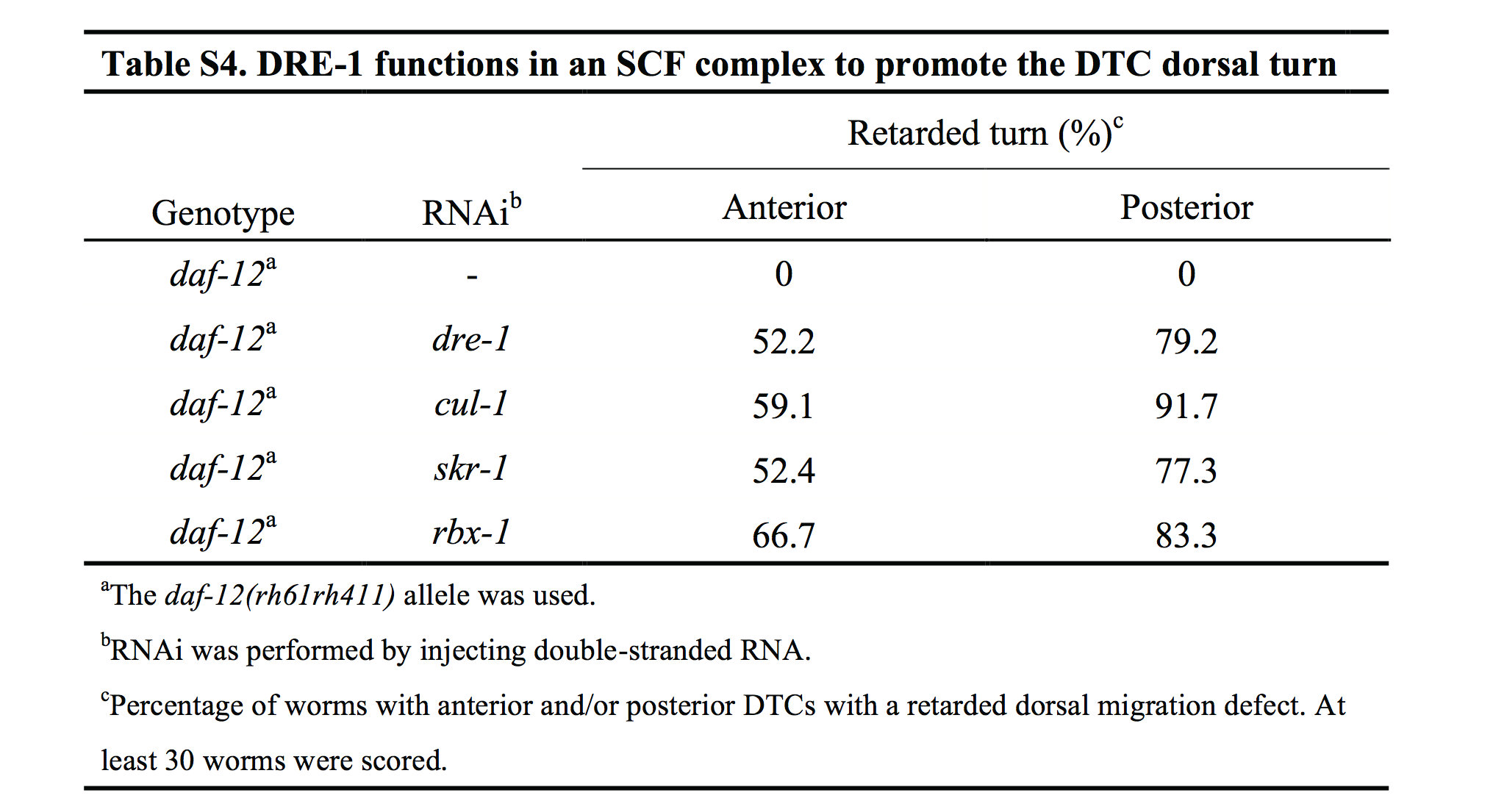

Supplement: Table S4 — DRE-1 functions in an SCF complex to promote the DTC dorsal turn. (TIFF) [file pgen.1004428.s009.tiff]

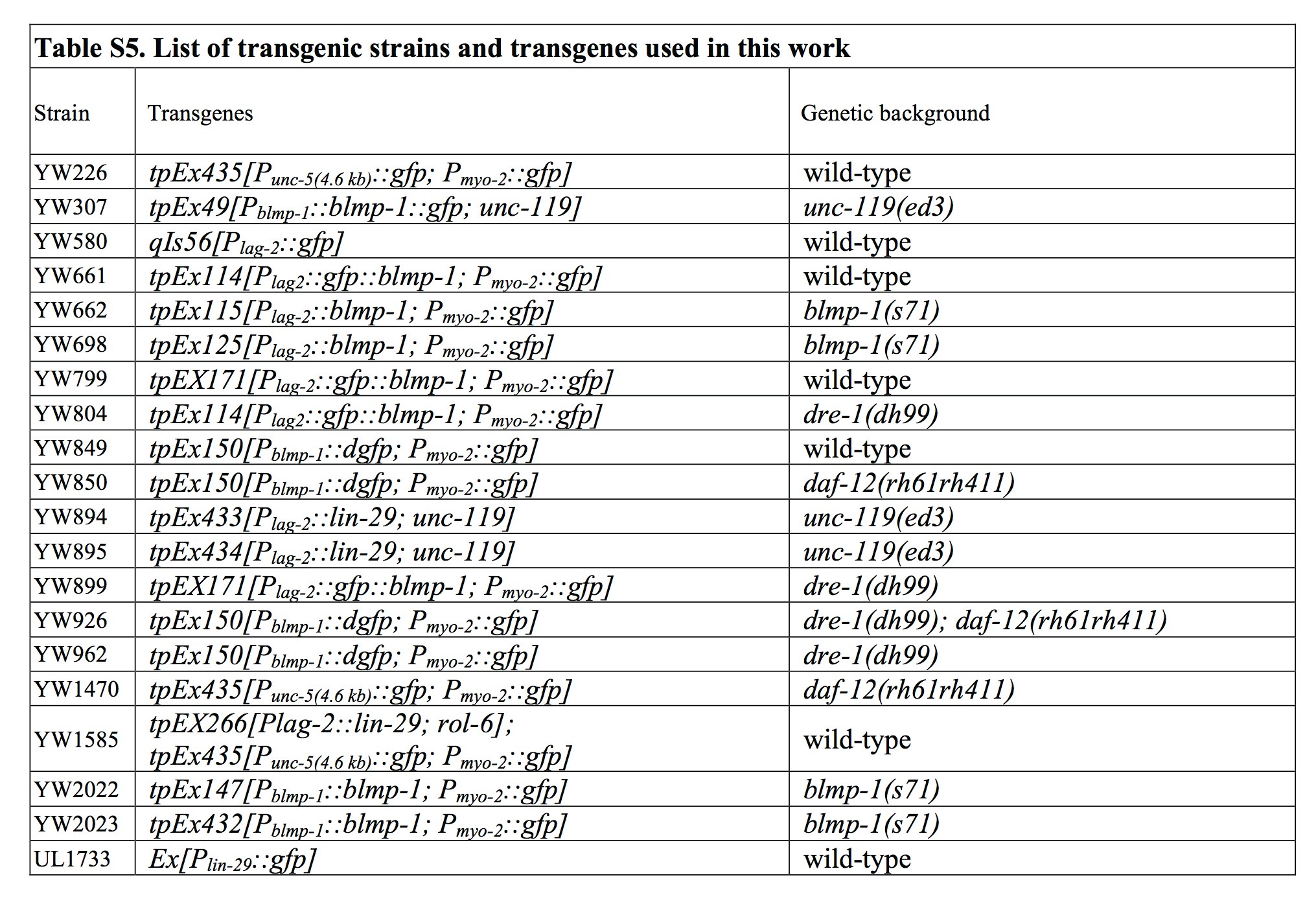

Supplement: Table S5 — List of transgenic strains and transgenes used in this work. (TIFF) [file pgen.1004428.s010.tiff]

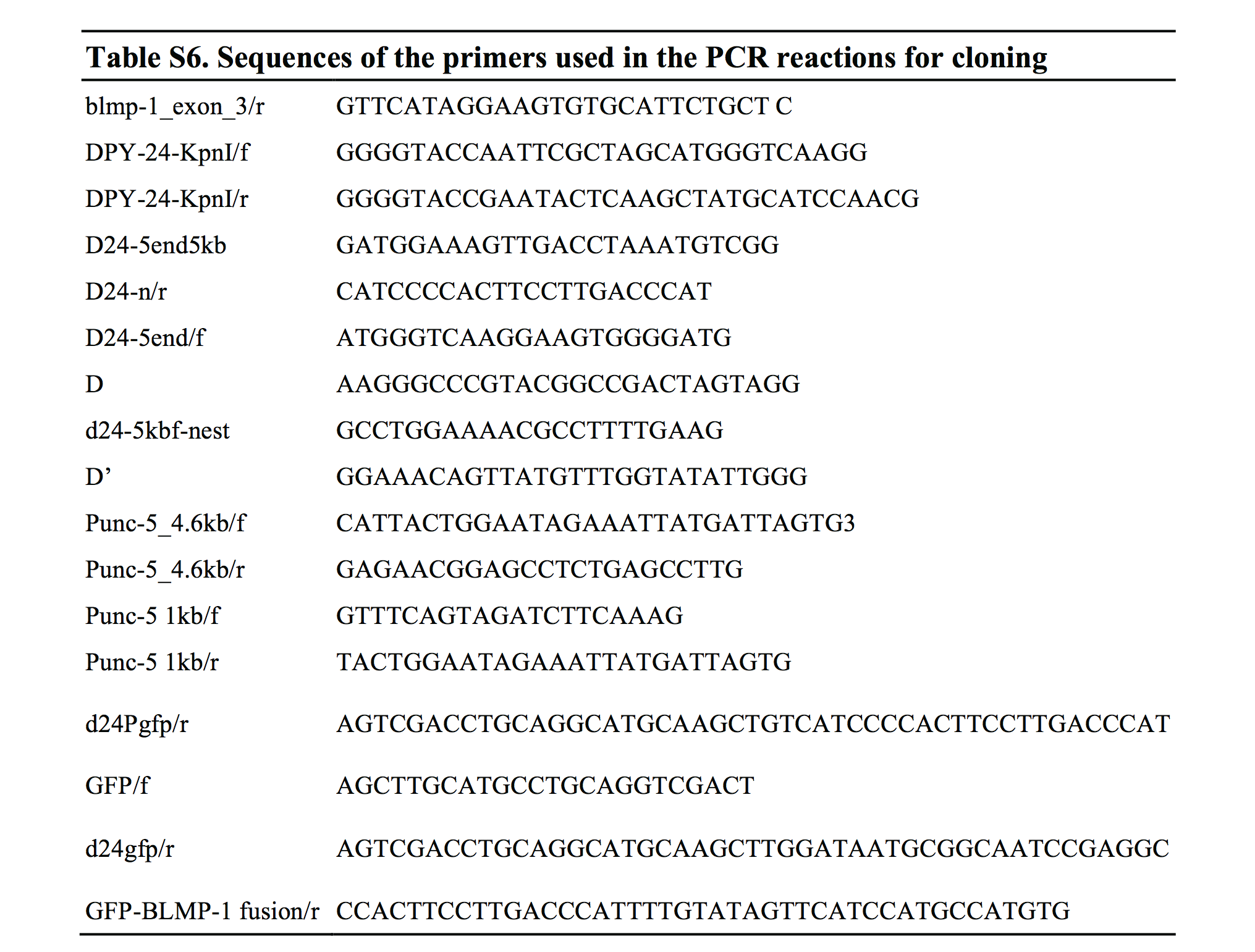

Supplement: Table S6 — Sequences of the primers used in the PCR reactions for cloning. (TIFF) [file pgen.1004428.s011.tiff]
